# Comparative effectiveness of pharmacotherapy for heart failure with preserved ejection fraction: A systematic review and network meta‐analysis

**DOI:** 10.1111/dom.70503

**Published:** 2026-01-26

**Authors:** Szu‐Han Chen, Yu‐Wen Tseng, Chi‐Jung Huang, Shu‐Mei Yang, Marat Fudim, Shao‐Yuan Chuang, Shih‐Hsien Sung, Hao‐Min Cheng

**Affiliations:** ^1^ School of Medicine, College of Medicine National Yang Ming Chiao Tung University Taipei Taiwan; ^2^ School of Chinese Medicine, College of Medicine National Yang Ming Chiao Tung University Taipei Taiwan; ^3^ Division of Evidence‐based Medicine, Department of Medical Education Taipei Veterans General Hospital Taipei Taiwan; ^4^ Division of Faculty Development, Department of Medical Education Taipei Veterans General Hospital Taipei Taiwan; ^5^ Department of Medicine Duke University Medical Center Durham North Carolina USA; ^6^ Duke Clinical Research Institute Durham North Carolina USA; ^7^ Division of Preventive Medicine and Health Service Research Institute of Population Health Sciences, National Health Research Institutes Miaoli Taiwan; ^8^ Cardiovascular Research Center, College of Medicine National Yang Ming Chiao Tung University Taipei Taiwan; ^9^ Division of Cardiology, Department of Medicine Taipei Veterans General Hospital Taipei Taiwan; ^10^ Institute of Emergency and Critical Care Medicine, College of Medicine National Yang Ming Chiao Tung University Taipei Taiwan; ^11^ General Clinical Research Center Taipei Veterans General Hospital Taipei Taiwan; ^12^ PhD Program of Interdisciplinary Medicine (PIM) National Yang Ming Chiao Tung University College of Medicine Taipei Taiwan; ^13^ Institute of Public Health and Community Medicine Research Centre National Yang Ming Chiao Tung University School of Medicine Taipei Taiwan

**Keywords:** GLP‐1 receptor agonist, heart failure with preserved ejection fraction, updated network meta‐analysis

## Abstract

**Aim:**

Heart failure with preserved ejection fraction (HFpEF) presents a therapeutic challenge, characterised by a paucity of validated treatments. Emerging data suggest that targeting adiposity is central to HFpEF pathogenesis. We conducted an updated network meta‐analysis to compare the efficacy of emerging and established HFpEF therapies.

**Materials and Methods:**

We systematically searched PubMed, Embase and Cochrane Library from inception to April 2025 for randomised controlled trials enrolling patients with HFpEF and evaluating pharmacotherapies, including angiotensin‐converting enzyme inhibitors, angiotensin receptor blockers, beta blockers, mineralocorticoid receptor antagonists (MRAs), digoxin, angiotensin receptor‐neprilysin inhibitor, sodium‐glucose transporter 2 inhibitors (SGLT2is), glucagon‐like peptide‐1 receptor agonists (GLP‐1 RAs), nitrates and nitrites. The primary outcome was a composite of cardiovascular death and heart failure (HF) hospitalisation. The secondary outcomes included cardiovascular death, all‐cause mortality, worsening HF events, change in the 6‐min walk test (6MWT) distance, Kansas City Cardiomyopathy Questionnaire Clinical Summary Score (KCCQ‐CSS) and N‐terminal pro‐B‐type natriuretic peptide levels. A frequentist random‐effects NMA was conducted.

**Results:**

Thirty‐nine trials with 78 treatment arms and 48 235 patients were enrolled. Compared with placebo, GLP‐1 RAs (HR: 0.73, 95% CI 0.61–0.88) and SGLT2is (HR: 0.79, 95% CI 0.70–0.90; P‐score: 0.807) significantly reduced the risk of cardiovascular death and HF hospitalisation. GLP‐1 RAs showed the highest probability of ranking first (P‐score: 0.871). GLP‐1 RAs elicited the greatest improvement in functional outcomes, including the 6MWT (mean difference: +17.60 m, 95% CI 8.53–26.67) and KCCQ‐CSS (mean difference: +7.38 points, 95% CI 5.51–9.26). No statistically significant differences in cardiovascular death or all‐cause mortality were observed among the treatments.

**Conclusions:**

In patients with HFpEF, GLP‐1RA, SGLT2i and MRA significantly reduced the risk of cardiovascular death and HF hospitalisation, while GLP‐1RA additionally improved the functional and quality‐of‐life outcomes. GLP‐1RA and SGLT2i significantly reduced HF morbidity, and GLP‐1RA uniquely improved functional status, positioning adiposity modulation as a central therapeutic target in HFpEF.

## 1 INTRODUCTION

Heart failure with preserved ejection fraction (HFpEF) accounts for approximately half of all heart failure (HF) cases and is increasingly prevalent among older adults, women and individuals with hypertension, diabetes or obesity.^1^, ^2^, ^3^, ^4^, ^5^, ^6^ Although HFpEF has a lower short‐term mortality than HF with reduced ejection fraction (HFrEF), it remains associated with high hospitalisation rates and poor long‐term outcomes. Its clinical management is complicated by heterogeneous pathophysiology and variable definitions of preserved ejection fraction, limiting comparability across trials and therapeutic consistency.^7^, ^8^ Emerging data suggest that targeting adiposity is central to HFpEF pathogenesis. The association between diabetes and HFpEF is mediated primarily through visceral adiposity,^9^ with GLP‐1 RAs exerting benefits by alleviating adipose‐driven inflammation and cardiac steatosis.

Over the past two decades, numerous randomised controlled trials (RCTs) have evaluated pharmacologic therapies for HFpEF. The CHARM‐Preserved trial found that candesartan modestly reduced HF hospitalisations without impacting cardiovascular (CV) mortality.^10^ PARAGON‐HF showed a non‐significant benefit of sacubitril/valsartan versus valsartan, with possible effects in women and those with lower EF.^11^ In contrast, sodium‐glucose co‐transporter 2 inhibitors (SGLT2is) have demonstrated more robust outcomes: EMPEROR‐Preserved (empagliflozin) and DELIVER (dapagliflozin) both significantly reduced the composite of CV death or HF hospitalisation.^12^, ^13^ Recently, glucagon‐like peptide‐1 receptor agonists (GLP‐1 RAs) have emerged as promising agents, particularly for obesity‐related HFpEF. STEP‐HFpEF and STEP‐HFpEF DM trials demonstrated substantial reductions in HF‐related outcomes, although these agents remain underrepresented in current guidelines.^3^, ^14^, ^15^, ^16^ Previous network meta‐analyses (NMAs) preceded the publication of STEP‐HFpEF and FINEARTS‐HF; our analysis integrates these and other recent trials to redefine pharmacologic hierarchy in HFpEF. These observations necessitate an updated NMA to compare therapies across clinical and patient‐centred outcomes in HFpEF, especially the novel emerging treatment options.

## 2 MATERIALS AND METHODS

We performed a systematic review and NMA that conformed with the Preferred Reporting Items for Systematic Reviews and Meta‐Analyses (PRISMA) extension statement for NMAs.^17^ The predetermined research protocol was officially published in PROSPERO (registration number: CRD420251020182).^18^

### 2.1 Searching strategy

A comprehensive search of the PubMed, Cochrane Central Register of Controlled Trials (CENTRAL) and Embase databases was conducted from inception to April 2025 for eligible RCTs. The detailed search strategy is outlined in the Supplemental Appendix.

We included studies that met the following criteria: (1) RCTs or post‐hoc analyses of RCTs; (2) study population comprising adult patients with HFpEF, which was defined as a left ventricular ejection fraction (LVEF) greater than or equal to 40%; and (3) methodology entailing comparison of the efficacy or safety of any of the following pharmacological agents: angiotensin‐converting enzyme inhibitors (ACEis), angiotensin receptor blockers (ARBs), beta blockers (BBs), mineralocorticoid receptor antagonists (MRAs), digoxin, angiotensin receptor‐neprilysin inhibitor (ARNI), SGLT2is, GLP‐1 RAs, nitrates or nitrites. Studies were excluded if they enrolled patients with predominantly reduced LVEF (<40%), did not report the relevant outcomes or involved non‐pharmacological interventions exclusively.

The primary outcome was a composite of the time to CV death and first hospitalisation for HF (HHF). The secondary outcomes included CV death, all‐cause mortality, worsening HF events, change in the 6‐min walk test (6MWT), Kansas City Cardiomyopathy Questionnaire Clinical Summary Score (KCCQ‐CSS) and N‐terminal pro‐B‐type natriuretic peptide (NT‐proBNP) levels.

### 2.2 Data extraction and quality assessment

Data from eligible studies were extracted independently by two reviewers (SHC and YWT) using a standardised data collection form. Extracted variables included study characteristics, patient demographics, intervention and comparator details, outcome measures and follow‐up duration. For trials reporting outcomes at multiple time points, we prioritised the longest available follow‐up. The risk of bias for each included study was assessed using the Cochrane Risk of Bias 2.0 tool. Grading of recommendations assessment, development and evaluation (GRADE) for NMA was used to assess the certainty of evidence.^19^ Disagreements during data extraction or quality assessment were resolved through discussion or by consultation with a third reviewer (HMC). To evaluate potential publication bias and the presence of small‐study effects, Egger's regression test was applied to the pairwise treatment effects of studies contributing to the main network.

### 2.3 Strategy for data synthesis

We conducted a NMA within a frequentist framework using the netmeta package in R (version 4.5.1). A random‐effects model was employed to account for between‐study heterogeneity. We estimated the relative treatment effects using hazard ratios (HRs) for time‐to‐event outcomes and mean differences (MDs) for continuous outcomes, each with 95% confidence intervals (CIs).

The network structure was constructed using direct and indirect comparisons across all included interventions. Transitivity was assessed by comparing the distributions of potential effect modifiers (e.g., age, sex, LVEF) across treatment comparisons. Statistical consistency between direct and indirect evidence was evaluated globally using the design‐by‐treatment interaction model and locally via node‐splitting methods. P‐scores were used to rank treatments based on their relative effectiveness or safety. All statistical analyses were performed using R software (R Foundation for Statistical Computing). A two‐sided *P*‐value < 0.05 was considered statistically significant.

## 3 RESULTS

**FIGURE 1:**
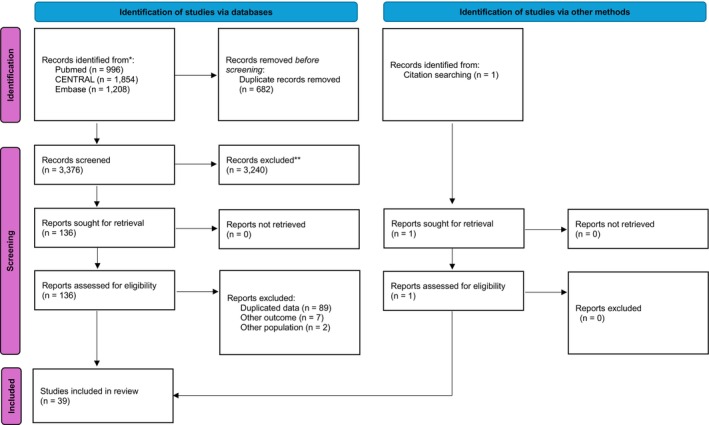
PRISMA flowchart. Flowchart illustrating the process of study identification, screening, eligibility assessment and inclusion in the network meta‐analysis.

A total of 39 RCTs enrolling 48 235 patients with HFpEF were included in the NMA. The study selection process is depicted in the PRISMA flow diagram (Figure 1). All included studies enrolled adult patients with an LVEF ≥40% and compared at least one of the following pharmacological treatments: ACEi, ARB, BB, MRA, digoxin, ARNI, SGLT2i, nitrates/nitrites and GLP‐1 RA. The characteristics of the included studies are summarised in Table S1 and can be visually confirmed in Figure S1.

### 3.1 Quality assessment

Risk of bias assessment revealed that 92.1% of the trials included were judged as having either a low risk or some concerns by the Cochrane Risk of Bias 2.0 tool (Figure S3). No significant evidence of publication bias was detected for most outcomes, including the primary composite outcome (*p* = 0.1847), CV death (*p* = 0.8925), all‐cause mortality (*p* = 0.8004), worsening HF (*p* = 0.1473), 6MWT change (*p* = 0.3184) and NT‐proBNP change (*p* = 0.2003). A notable small‐study effect, likely associated with publication bias, was detected for the change in the KCCQ‐CSS (*p* = 0.0375).

### 3.2 Assessment of network meta‐analysis assumptions

The similarity assumption was assessed by comparing baseline patient characteristics across treatment comparisons (Table S1 and Figure S1). Overall, the distribution of baseline characteristics was comparable, except for trials evaluating GLP‐1 RA, which predominantly enrolled patients with a high body mass index (BMI). The homogeneity assumption was evaluated using the I^2^ statistic for each pairwise comparison. All outcomes demonstrated low statistical heterogeneity (*I* ^2^ = 0%), supporting the assumption of homogeneity. The consistency assumption was examined using node‐splitting analyses. For all outcomes, the *p*‐values comparing direct and indirect estimates were greater than 0.05, indicating no evidence of inconsistency and supporting the validity of the consistency assumption.

### 3.3 Primary outcome

The primary outcome was a composite of time to CV death or first HHF. Among the 39 included trials, this outcome was reported in 12 studies encompassing a total of 22 316 patients.

**FIGURE 2:**
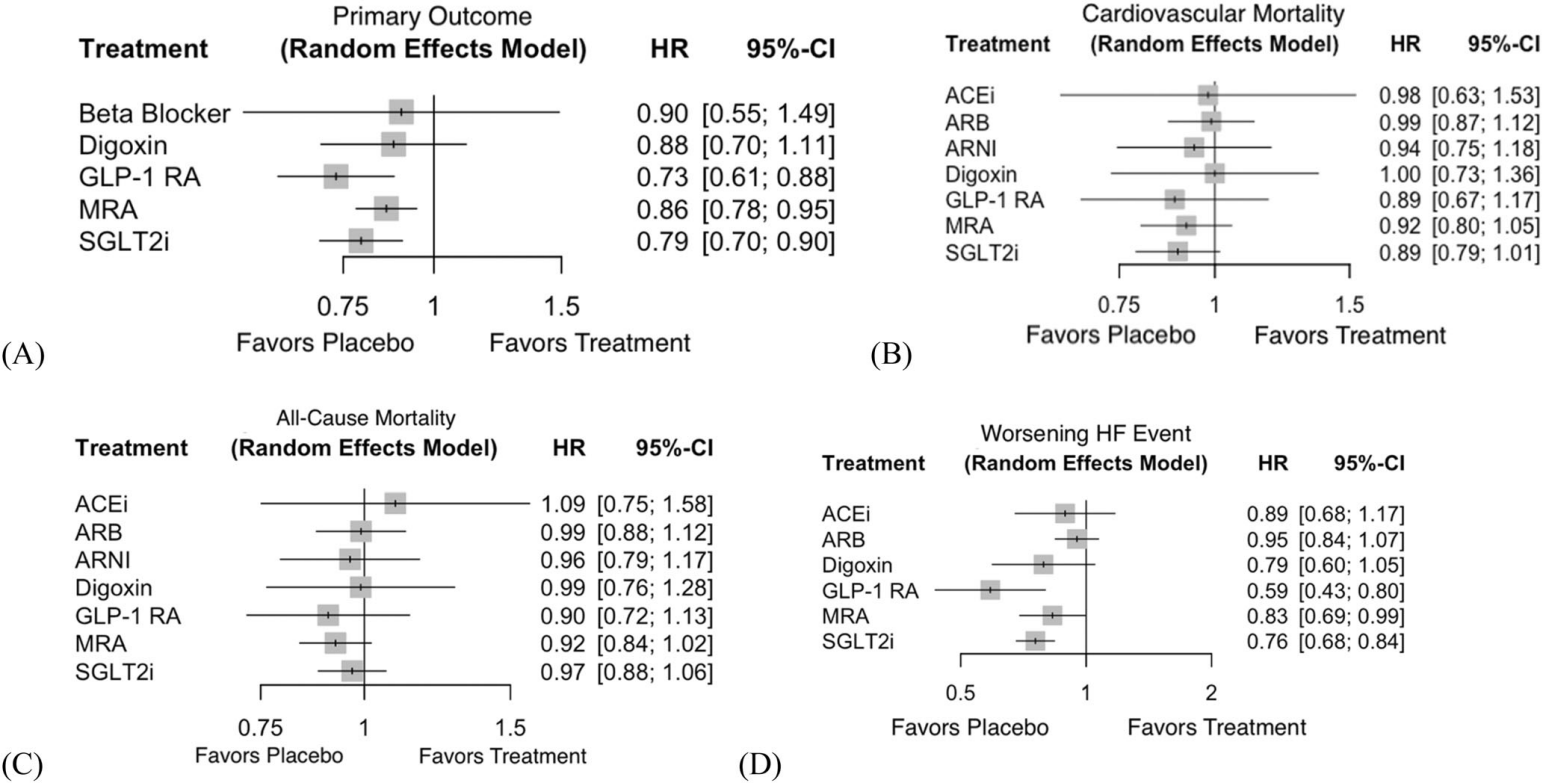
Forest plot of mortality and worsening heart failure event–related outcome. Forest plots showing the HRs for (A) the primary outcome, (B) cardiovascular mortality, (C) all‐cause mortality and (D) worsening HF event compared with placebo. ACEi, angiotensin‐converting enzyme inhibitor; ARB, angiotensin receptor blocker; ARNI, angiotensin receptor‐neprilysin inhibitor; CI, confidence interval; GLP‐1 RA, glucagon‐like peptide‐1 receptor agonist; HR, hazard ratio; MRA, mineralocorticoid receptor antagonist; SGLT2i, sodium‐glucose transporter 2 inhibitors.

As shown in Figure 2, compared with placebo, both GLP‐1 RA and SGLT2i significantly reduced the risk of CV death or first HHF. Specifically, GLP‐1 RAs showed the greatest reduction in the risk of CV death or first HHF (HR 0.73, 95% CI 0.61–0.88), followed by SGLT2is (HR 0.79, 95% CI 0.70–0.90). MRAs were also associated with a modest but statistically significant benefit (HR 0.86, 95% CI 0.78–0.95), while digoxin (HR 0.88, 95% CI 0.70–1.11) and beta blockers (HR 0.90, 95% CI 0.55–1.49) failed to yield statistically significant improvements.

The treatment ranking probability obtained from P‐scores using the random‐effects model (Table S2) — where a higher P‐score indicates a higher probability of being the most effective treatment — indicated that GLP‐1RA was the most effective intervention (P‐score = 0.8705), followed by SGLT2i (P‐score = 0.7084), MRA (P‐score = 0.4749), digoxin (P‐score = 0.4320), beta blockers (P‐score = 0.4173) and placebo (P‐score = 0.0967). Overall, the GRADE assessment yielded moderate certainty of evidence for the primary composite outcome, mainly due to imprecision in several smaller comparisons despite generally low risk of bias and consistent network estimates. (Table S4).

### 3.4 CV and all‐cause mortality

No treatment was associated with a significant reduction in CV death compared with placebo. The pooled HRs (95% CI) were 0.89 (0.67–1.17) for GLP‐1 RA, 0.89 (0.79–1.01) for SGLT2i and 0.92 (0.80–1.05) for MRA. Other treatments, including ACEi, ARB, ARNI and digoxin, also showed non‐significant associations, with HRs ranging from 0.94 to 1.00.

Similarly, no significant reduction in all‐cause mortality was observed for any of the evaluated treatments. The HRs (95% CI) for GLP‐1 RA, SGLT2i and MRA were 0.90 (0.72–1.13), 0.97 (0.88–1.06) and 0.92 (0.84–1.02), respectively. Other agents, including digoxin (HR: 0.99, 95% CI: 0.76–1.28) and ARNI (HR: 0.96, 0.79–1.17), failed to show any significant benefit.

P‐score rankings suggested that MRA and GLP‐1 RA possessed the highest probability of being the most effective drugs in reducing all‐cause mortality (P‐scores: 0.7238 and 0.7116), while SGLT2i showed the highest effectiveness in preventing CV death (0.7063), followed closely by GLP‐1 RA (0.6650).

### 3.5 Worsening HF events

Both GLP‐1 RA and SGLT2i were significantly associated with a lower risk of worsening HF events compared with placebo. GLP‐1 RA demonstrated the strongest effect (HR: 0.59, 95% CI: 0.43–0.80), followed by SGLT2i (HR: 0.76, 95% CI: 0.68–0.84). MRA also showed a modest but statistically significant benefit (HR: 0.83, 95% CI: 0.69–0.99). In contrast, ACEi, ARB and digoxin did not significantly reduce the risk of worsening of HF, with HRs ranging from 0.79 to 0.95.

The ranking probabilities further supported the superiority of GLP‐1 RA, which had the highest P‐score (0.9662), followed by SGLT2i (0.7252) and digoxin (0.6054). These findings suggest that GLP‐1 RA and SGLT2i are not only beneficial for the composite outcomes but may also offer meaningful protection against recurrent or progressive HF episodes in patients with LVEF ≥40%.

### 3.6 Functional and quality‐of‐life‐related outcomes

**FIGURE 3:**
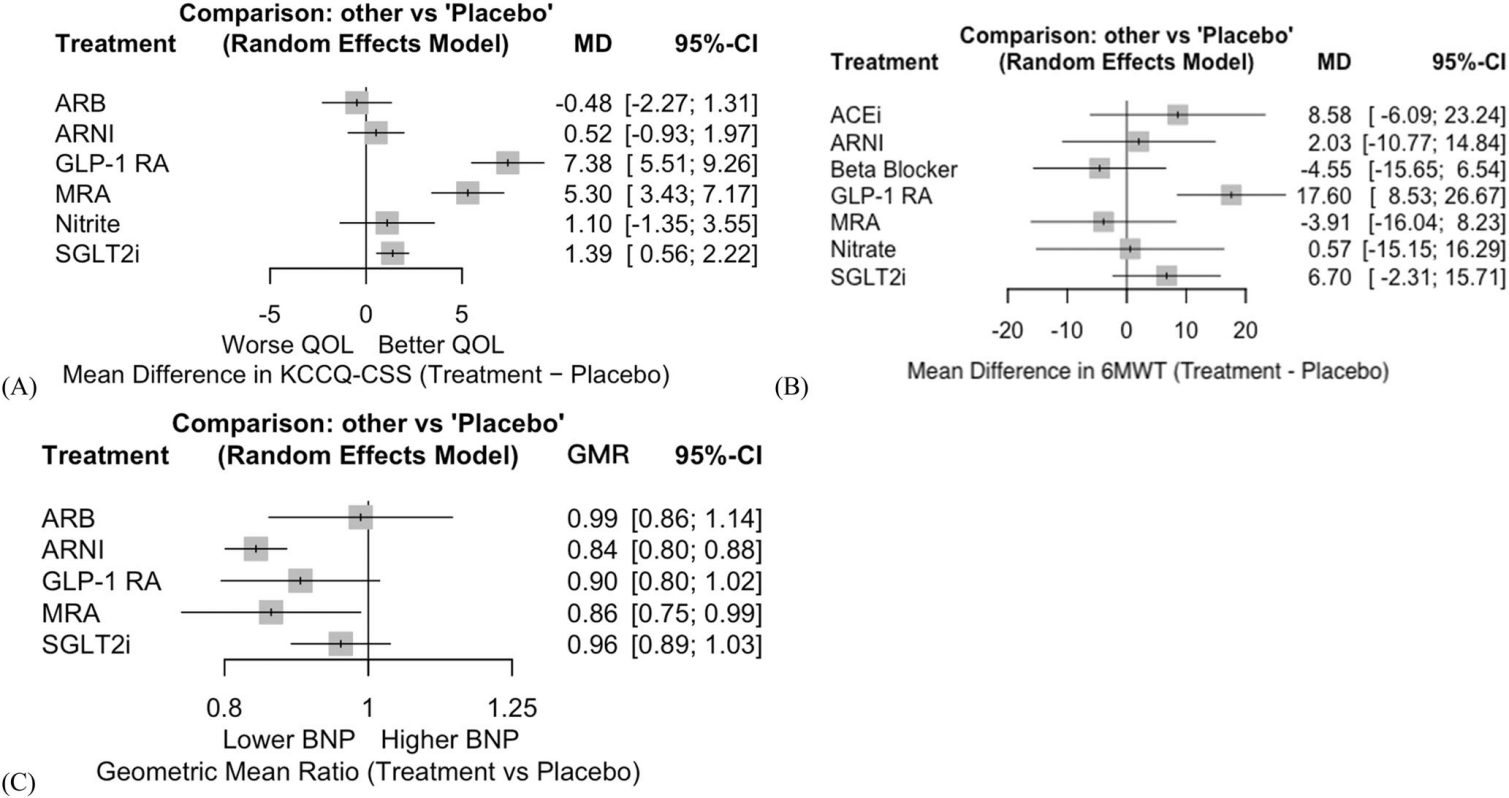
Forest plot of functional and quality‐of‐life‐related outcomes. Forest plots showing the mean difference for (A) KCCQ‐CSS and (B) 6MWT compared with placebo, and the (C) geometric mean ratio of NT‐proBNP level compared with placebo. 6MWT, 6‐minute walk test; ACEi, angiotensin‐converting enzyme inhibitor; ARB, angiotensin receptor blocker; ARNI, angiotensin receptor‐neprilysin inhibitor; CI, confidence interval; GLP‐1 RA, glucagon‐like peptide‐1 receptor agonist; GMR, geometric mean ratio; KCCQ‐CSS, Kansas City Cardiomyopathy Questionnaire Clinical Summary Score; MD, mean difference; MRA, mineralocorticoid receptor antagonist; NT‐proBNP, N‐terminal pro‐B‐type natriuretic peptide; SGLT2i, sodium‐glucose transporter 2 inhibitors.

Among the functional outcomes shown in Figure 3, GLP‐1 RA consistently demonstrated the most pronounced improvements. GLP‐1 RA, which was associated with a significant increase in walking distance on the 6MWT compared to placebo [mean difference (MD): +17.60 m; 95% CI: 8.53–26.67], ranked the highest among all interventions (P‐score: 0.9624). None of the other treatment options showed a significant gain.

In terms of the patient‐reported quality of life, GLP‐1 RAs ranked the highest, yielding the largest improvement in the KCCQ‐CSS compared with placebo (MD: +7.38 points; 95% CI: 5.51–9.26). MRA and SGLT2i also provided significant benefits (MRA: +5.30; 95% CI: 3.43–7.17; SGLT2i: +1.39; 95% CI: 0.56–2.22), with respective P‐scores of 0.8430 and 0.5665. In contrast, ARB and ARNI showed minimal or no improvement in the KCCQ‐CSS.

Overall, GLP‐1 RA emerged as the most favourable option for enhancing the exercise capacity and health‐related quality of life, with consistent superiority across functional domains.

### 3.7 Change in NT‐proBNP levels

Among interventions with available data on the change in NT‐proBNP levels (Figure 3), ARNI and MRA were associated with significant reductions compared with placebo [ARNI: geometric mean ratio (GMR) 0.84, 95% CI: 0.80–0.88; MRA: GMR 0.86, 95% CI: 0.75–0.99]. GLP‐1 RA and SGLT2i showed modest, non‐significant effects.

### 3.8 Sensitivity analysis

To further assess the treatment effects among individuals with obesity, we performed a restricted NMA including only trials enrolling participants with a mean baseline BMI ≥30 kg/m^2^. Under this constraint, three GLP‐1 RA trials and one MRA trial were eligible for the analysis of the primary outcome. The pooled hazard ratios were 0.58 (95% CI: 0.40–0.84) for GLP‐1 RA and 0.89 (95% CI: 0.77–1.03) for MRA. The P‐score for GLP‐1 RA was 0.9901, indicating consistent superiority of GLP‐1 RA in treating HFpEF in this subgroup with obesity.

## 4 DISCUSSION

In this comprehensive NMA including 48 235 patients across 39 trials, we found that GLP‐1 RAs and SGLT2 inhibitors significantly reduced the composite of cardiovascular death and HHF compared with placebo, with GLP‐1 RAs ranking highest overall. GLP‐1 RAs also provided the greatest improvements in functional outcomes such as 6MWT and KCCQ‐CSS, underscoring their unique patient‐centred benefits. These results complement the robust outcome data of SGLT2 inhibitors and extend evidence for the emerging role of GLP‐1 RAs in HFpEF.

Our findings are consistent with recent randomised trials, including STEP‐HFpEF, STEP‐HFpEF DM and SUMMIT, all of which demonstrated that metabolic interventions with GLP‐1–based agents improved symptoms, exercise capacity and quality of life in patients with obesity‐related HFpEF. Together, these results and our NMA highlight GLP‐1 RAs as a phenotype‐specific therapy rather than a ‘one‐size‐fits‐all’ approach for HFpEF. The inclusion of these obesity‐focused trials materially reshapes the previous treatment hierarchy, emphasising the central role of metabolic dysfunction and adiposity in HFpEF pathophysiology. Mechanistically, recent evidence suggests that excess visceral adiposity—through adipokine dysregulation, sodium retention and cardiac steatosis—is a key driver of diabetes‐associated HFpEF, rather than hyperglycaemia alone.^9^

This updated analysis materially changes prior therapeutic rankings by incorporating GLP‐1 RA data, which were absent from all earlier NMAs.^20^, ^21^, ^22^, ^23^ As summarised in Table S5, our review is the first to integrate all modern pharmacologic classes—including GLP‐1 RA and SGLT2i—within a unified HFpEF‐specific network meta‐analytic framework with formal functional outcomes assessment. We included comprehensive and emerging pharmacologic classes, namely nitrates, nitrites and GLP‐1 RAs, which were absent in earlier comparisons, thereby providing a more comprehensive view of the current therapeutic options. Importantly, we placed a robust emphasis on the patient‐centred functional outcomes, including the 6MWT and KCCQ‐CSS, which reflect exercise capacity and quality of life, respectively. This update aligns with a growing consensus in the field of HF that traditional endpoints such as mortality and hospitalisation, albeit important, may not fully capture treatment benefits from the patient's perspective.^24^, ^25^, ^26^ Previous studies have emphasised that improving daily functioning is central to therapeutic success, especially given that patients with HFpEF often face chronic physical limitations without imminent risk of death.^27^ By incorporating these functional endpoints, our analysis offers a more holistic evaluation of the therapeutic value of pharmacological agents and responds to the shifting clinical and regulatory landscape that increasingly prioritises patient‐reported and function‐based outcomes. Although NT‐proBNP reduction may reflect attenuation of cardiac stress, its clinical interpretation is limited by high variability and susceptibility to confounding factors, such as obesity.^28^ As such, its role as a surrogate endpoint in contemporary HF research remains secondary to patient‐centred outcomes.^29^

GLP‐1 RAs demonstrated consistent benefits across clinical and functional endpoints, despite limited prior evidence in HFpEF. Unlike conventional HF therapies primarily developed for HFrEF, GLP‐1 RAs were designed to target cardiometabolic risk in patients with obesity or diabetes—key drivers of HFpEF pathophysiology.^14^, ^30^, ^31^, ^32^ This may explain their greater improvements in quality of life and exercise capacity. However, most trials enrolled patients with high BMI, limiting generalisability to non‐obese HFpEF, and the overall evidence base remains smaller than that of other drug classes. Potential limitations of GLP‐1 RAs should also be acknowledged. Emerging evidence suggests that weight loss induced by GLP‐1 RAs may be accompanied by reductions in lean body mass, raising concerns regarding sarcopenia or muscle wasting, particularly in older patients with HFpEF who are already vulnerable to frailty and impaired exercise tolerance.^33^, ^34^ In parallel, our findings reaffirm the efficacy of SGLT2 inhibitors in reducing HF‐related morbidity, consistent with large‐scale trials such as EMPEROR‐Preserved and Preserved‐HF. Although their effect size appeared smaller than that of GLP‐1 RAs, SGLT2is are supported by broader, more definitive data, underscoring the complementary strengths of these two therapeutic classes. In contrast to heart failure–related outcomes, available evidence for stroke and atrial fibrillation in HFpEF remains limited and consistently neutral across drug classes (Table S7, Figures S5 and S6), underscoring the need for adequately powered trials focusing on these endpoints.

HFpEF commonly coexists with systemic inflammation, insulin resistance and visceral adiposity^35^, ^36^, ^37^, ^38^—pathways directly targeted by GLP‐1 receptor agonists.^39^, ^40^ Beyond glucose lowering and weight reduction, GLP‐1 RAs attenuate inflammatory signalling and reduce circulating cytokines such as interleukin‐6, tumour necrosis factor‐α and high‐sensitivity C‐reactive protein, thereby improving endothelial function and myocardial remodelling.^41^, ^42^, ^43^, ^44^ These effects are particularly relevant in obesity‐related HFpEF, where visceral adiposity drives low‐grade inflammation and diastolic dysfunction. Collectively, the pleiotropic metabolic and anti‐inflammatory actions of GLP‐1 RAs may underlie the symptomatic and functional benefits observed in recent trials, supporting their role in metabolically driven HFpEF phenotypes.^14^, ^45^

While MRAs were analysed as a single class, pharmacological differences exist between steroidal (spironolactone, eplerenone) and non‐steroidal agents (finerenone).^46^ Among the included studies, six evaluated traditional steroidal MRAs and one investigated finerenone, recently tested in the FINEARTS‐HF trial.^47^ Unlike spironolactone, which showed inconsistent benefits in HFpEF (e.g., regional heterogeneity in TOPCAT),^48^ finerenone demonstrated favourable effects on KCCQ scores and natriuretic peptide levels, particularly in obesity‐ or metabolism‐related phenotypes. Notably, although finerenone significantly reduced the primary composite outcome (worsening HF or CV death) in FINEARTS‐HF, this benefit was largely driven by a reduction in worsening HF events, whereas no statistically significant reduction in CV mortality was observed. These findings suggest that the therapeutic benefit of finerenone in HFpEF predominantly reflects mitigation of HF progression rather than an effect on CV death.^47^ No evidence of a reduction in stroke was reported with finerenone in either FINEARTS‐HF or previous cardiovascular outcome trials including patients with chronic kidney disease and type 2 diabetes, including FIGARO‐DKD and FIDELIO‐DKD.^49^, ^50^ This limitation is clinically relevant, particularly in older patients with diabetes and/or chronic kidney disease who are at increased baseline risk for cerebrovascular events.^51^, ^52^ As only one finerenone trial was available, definitive comparisons between steroidal and non‐steroidal MRAs remain premature, underscoring the need for head‐to‐head and phenotype‐guided studies to clarify potential incremental benefits.^53^

Our findings support the inclusion of GLP‐1 RAs, alongside SGLT2is, as foundational therapy in phenotype‐specific management of HFpEF. Contemporary guidelines increasingly recognise the heterogeneity of HFpEF and the limited efficacy of traditional agents.^2^, ^3^ In this context, GLP‐1 RAs provide consistent benefits across symptoms, functional capacity and quality of life, complementing the established effects of SGLT2is. These results highlight the need for future recommendations to adopt a more tailored, goal‐oriented framework that integrates metabolic and patient‐centred outcomes into individualised treatment algorithms.^54^, ^55^, ^56^, ^57^

This study offers several strengths that enhance the validity and relevance of its findings. First, it represents the most updated and comprehensive NMA of pharmacological therapies for HFpEF to date, incorporating not only established drug classes but also emerging agents such as GLP‐1 RAs, nitrates and nitrites. Second, by including a broad range of both clinical and functional endpoints, including CV outcomes, worsening HF events, 6MWT and KCCQ‐CSS, we provide a more holistic appraisal of therapeutic value that aligns with evolving priorities in HF care. Third, the application of a frequentist network meta‐analytic framework with rigorous assessment of transitivity and consistency strengthened the credibility of the indirect comparisons and treatment rankings.

Nevertheless, important limitations should be acknowledged. First, although GLP‐1 RAs demonstrated consistent superiority across multiple domains, the evidence was predominantly derived from trials enrolling individuals with obesity or metabolic syndrome, which limits the generalisability of our findings to non‐obese HFpEF populations. Future RCTs with broader inclusion criteria and longer follow‐up are needed to confirm the durability of benefits and assess potential synergistic strategies combining GLP‐1 RAs and SGLT2is. Second, heterogeneity in trial populations, diagnostic criteria (e.g., LVEF thresholds) and outcome definitions may introduce between‐study variability and affect the comparability of the treatment effects. Third, the number of trials and sample sizes varied substantially across drug classes, with some comparisons being supported by limited data. Additionally, trial‐level meta‐analyses are inherently constrained by the lack of individual patient data, preventing detailed subgroup analyses and adjustment for covariates. Finally, differences in follow‐up durations across studies may affect outcome comparability, particularly for endpoints such as mortality.

## 5 CONCLUSIONS

The NMA indicates that GLP‐1 RA, SGLT2 and MRA reduce cardiovascular mortality or HHF in HFpEF. Further, GLP‐1 RA provide consistent clinical and functional benefits in HFpEF and should be evaluated as a disease‐modifying therapy, particularly in obesity‐related phenotypes.

## CONFLICT OF INTEREST STATEMENT

The authors declare no conflicts of interest.

## Supporting information


**Data S1.** Supporting information.

## Data Availability

This meta‐analysis used only data extracted from publicly available RCTs. All data are available in the published articles cited in the manuscript.
