# Supplementary material for: Comparative effectiveness of pharmacotherapy for heart failure with preserved ejection fraction: A systematic review and network meta‐analysis
Source: Diabetes Obes Metab. 2026 Jan 26;28(4):3137–45. doi: 10.1111/dom.70503 (PMC12992183; doi:10.1111/dom.70503)

Supplementary Appendix. Searching Strategy

**Pubmed:**

(heart failure with preserved ejection fraction OR HFpEF OR "Heart Failure, Diastolic"[Mesh] OR preserved ejection fraction)

AND

("Angiotensin-Converting Enzyme Inhibitors"[Mesh] OR ACEi OR ACEis OR ACE-I OR ACE inhibitor OR ACE inhibitors OR benazepril OR captopril OR delapril OR enalapril OR fosinopril OR quinapril OR imidapril OR lisinopril OR moexipril OR ramipril OR perindopril OR trandolapril

OR "Angiotensin Receptor Antagonists"[Mesh] OR angiotensin receptor blockers OR ARB OR irbesartan OR valsartan OR losartan OR candesartan OR olmesartan OR telmisartan OR eprosartan

OR "Adrenergic beta-Antagonists"[Mesh] OR beta blocker OR beta blockers OR β-blocker OR β-blockers OR alprenolol OR betaxolol OR bisoprolol OR bupranolol OR carvedilol OR carteolol OR celiprolol OR esmolol OR labetalol OR metoprolol OR nadolol OR nebivolol OR oxprenolol OR penbutolol OR pindolol OR practolol OR propranolol OR sotalol OR timolol OR acebutolol OR atenolol

OR "Mineralocorticoid Receptor Antagonists"[Mesh] OR MRA OR MRAs OR aldosterone receptor blocker OR aldosterone receptor blockers OR aldosterone blocker OR aldosterone blockers OR spironolactone OR eplerenone OR canrenone OR finerenone

OR "Digoxin"[Mesh]

OR angiotensin receptor neprilysin inhibitor OR angiotensin receptor neprilysin inhibitors OR angiotensin receptor-neprilysin inhibitor OR angiotensin receptor-neprilysin inhibitors OR ANRI OR sacubitril OR sacubitril-valsartan OR Entresto

OR "Sodium-Glucose Transporter 2 Inhibitors"[Mesh] OR SGLT-2 inhibitor OR SGLT-2 inhibitors OR SGLT2i OR SGLT2is OR canagliflozin OR empagliflozin OR dapagliflozin OR ertugliflozin OR sotagliflozin OR remogliflozin OR luseogliflozin OR ipragliflozin

OR “Nitrates"[Mesh] OR nitrate

OR "Glucagon-Like Peptide-1 Receptor Agonists"[Mesh] OR GLP-1 RA OR GLP-1 RAs OR albiglutide OR dulaglutide OR efpeglenatide OR exenatide OR iraglutide OR lixisenatide OR semaglutide OR tirzepatide)

AND

("randomized controlled trial"[Publication Type] OR "randomized"[tiab] OR “randomised"[tiab] OR "clinical trial"[Publication Type] )

**CENTRAL:**

#1: MeSH descriptor: [Heart Failure, Diastolic] explode all trees

#2: #1 OR heart failure with preserved ejection fraction OR HFpEF OR preserved ejection fraction

#3: MeSH descriptor: [Angiotensin-Converting Enzyme Inhibitors] explode all trees

#4: #3 OR ACEi OR ACEis OR ACE-I OR ACE inhibitor OR ACE inhibitors OR benazepril OR captopril OR delapril OR enalapril OR fosinopril OR quinapril OR imidapril OR lisinopril OR moexipril OR ramipril OR perindopril OR trandolapril

#5: MeSH descriptor: [Angiotensin Receptor Antagonists] explode all trees

#6: #5 OR angiotensin receptor blocker OR angiotensin receptor blockers OR ARB OR ARBs OR irbesartan OR valsartan OR losartan OR candesartan OR olmesartan OR telmisartan OR eprosartan

#7: MeSH descriptor: [Adrenergic beta-Antagonists] explode all trees

#8: #7 OR beta blocker OR beta blockers OR β-blocker OR β-blockers OR alprenolol OR betaxolol OR bisoprolol OR bupranolol OR carvedilol OR carteolol OR celiprolol OR esmolol OR labetalol OR metoprolol OR nadolol OR nebivolol OR oxprenolol OR penbutolol OR pindolol OR practolol OR propranolol OR sotalol OR timolol OR acebutolol OR atenolol

#9: MeSH descriptor: [Mineralocorticoid Receptor Antagonists] explode all trees

#10: #9 OR MRA OR MRAs OR aldosterone receptor blocker OR aldosterone receptor blockers OR aldosterone blocker OR aldosterone blockers OR spironolactone OR eplerenone OR canrenone OR finerenone

#11: MeSH descriptor: [Digoxin] explode all trees

#12: angiotensin receptor neprilysin inhibitor OR angiotensin receptor neprilysin inhibitors OR angiotensin receptor-neprilysin inhibitor OR angiotensin receptor-neprilysin inhibitors OR ANRI OR sacubitril OR sacubitril-valsartan OR Entresto

#13: MeSH descriptor: [Sodium-Glucose Transporter 2 Inhibitors] explode all trees

#14: #13 OR SGLT-2 inhibitor OR SGLT-2 inhibitors OR SGLT2i OR SGLT2is OR canagliflozin OR empagliflozin OR dapagliflozin OR ertugliflozin OR sotagliflozin OR remogliflozin OR luseogliflozin OR ipragliflozin

#15: MeSH descriptor: [Nitrates] explode all trees

#16: #15 OR nitrate OR nitrite

#17: MeSH descriptor: [Glucagon-Like Peptide-1 Receptor Agonists] explode all trees

#18: #15 OR GLP-1 RA OR GLP-1 RAs OR albiglutide OR dulaglutide OR efpeglenatide OR exenatide OR iraglutide OR lixisenatide OR semaglutide OR tirzepatide

#19: #2 AND (#4 OR #6 OR #8 OR 10 OR #11 OR #12 OR #14 OR #16 OR #18)

#20: "randomized controlled trial":ti,ab,kw

#21: clinical trial:ti,ab,kw

#22: #20 OR #21

#23: #19 AND #22

**Embase:**

('HF with preserved ejection fraction' OR 'HF-pEF (heart failure with preserved ejection fraction)' OR 'HFpEF (heart failure with preserved ejection fraction)' OR 'heart failure with preserved ejection fraction’)

AND

(('dipeptidyl carboxypeptidase inhibitor'/exp OR ACEi OR ACEis OR ACE-I OR ACE inhibitor OR ACE inhibitors OR benazepril OR captopril OR delapril OR enalapril OR fosinopril OR quinapril OR imidapril OR lisinopril OR moexipril OR ramipril OR perindopril OR trandolapril)

OR ('angiotensin receptor antagonist'/exp OR angiotensin receptor blockers OR ARB OR irbesartan OR valsartan OR losartan OR candesartan OR olmesartan OR telmisartan OR eprosartan)

OR ('beta adrenergic receptor blocking agent'/exp OR beta blocker OR beta blockers OR β-blocker OR β-blockers OR alprenolol OR betaxolol OR bisoprolol OR bupranolol OR carvedilol OR carteolol OR celiprolol OR esmolol OR labetalol OR metoprolol OR nadolol OR nebivolol OR oxprenolol OR penbutolol OR pindolol OR practolol OR propranolol OR sotalol OR timolol OR acebutolol OR atenolol)

OR ('mineralocorticoid antagonist'/exp OR MRA OR MRAs OR aldosterone receptor blocker OR aldosterone receptor blockers OR aldosterone blocker OR aldosterone blockers OR spironolactone OR eplerenone OR canrenone OR finerenone)

OR ('digoxin'/exp)

OR ('angiotensin receptor neprilysin inhibitor'/exp OR angiotensin receptor neprilysin inhibitor OR angiotensin receptor neprilysin inhibitors OR angiotensin receptor-neprilysin inhibitor OR angiotensin receptor-neprilysin inhibitors OR ANRI OR sacubitril OR sacubitril-valsartan OR Entresto)

OR ('sodium glucose cotransporter 2 inhibitor'/exp OR SGLT-2 inhibitor OR SGLT-2 inhibitors OR SGLT2i OR SGLT2is OR canagliflozin OR empagliflozin OR dapagliflozin OR ertugliflozin OR sotagliflozin OR remogliflozin OR luseogliflozin OR ipragliflozin)

OR ( 'nitrate'/exp OR nitrate:ti,ab OR 'nitrite'/exp OR nitrite:ti,ab)

OR ('glucagon like peptide 1 receptor agonist'/exp OR GLP-1 RA OR GLP-1 RAs OR albiglutide OR dulaglutide OR efpeglenatide OR exenatide OR iraglutide OR lixisenatide OR semaglutide OR tirzepatide))

AND

('randomized controlled trial'/exp OR 'clinical trial'/exp OR randomized:ti,ab OR randomised:ti,ab)

Supplementary Table S1. Baseline patient characteristics of all studies included

| Study | Intervention | Intervention_type | Comparator | Comparator_type | Intervention |  |  |  |  |  |  |  |  |  |  |  |  |  | Comparator |  |  |  |  |  |  |  |  |  |  |  |  |  | median follow-up | LVEF threshold |
| --- | --- | --- | --- | --- | --- | --- | --- | --- | --- | --- | --- | --- | --- | --- | --- | --- | --- | --- | --- | --- | --- | --- | --- | --- | --- | --- | --- | --- | --- | --- | --- | --- | --- | --- |
|  |  |  |  |  | Number | Age | Male% | Mean LVEF | Mean BMI | NYHA I | NYHA II | NYHA III | NYHA IV | eGFR | Hypertension | AF | DM | Prior MI | Number | Age | Male% | Mean LVEF | Mean BMI | NYHA I | NYHA II | NYHA III | NYHA IV | eGFR | Hypertension | AF | DM | Prior MI |  |  |
| Aldo-DHF | Spironolactone | MRA | Placebo | Placebo | 213 | 67 | 47.9% | 67.0% | 28.9 | 0 | 84.5% | 15.5% | 0.0% | 79 | 92.5% | 6.1% | 16.9% | N/A | 209 | 67 | 47.4% | 68.0% | 28.9 | 0.0% | 87.6% | 12.4% | 0.0% | 78.0 | 90.9% | 4.3% | 16.3% | N/A | 12 months | LVEF≥50% |
| Ancillary-DIG | Digoxin | Digoxin | Placebo | Placebo | 492 | 66.7 | 57.9% | 55.4% | N/A | 19.1% | 59.3% | 20.7% | 0.8% | 62.4 | 62.0% | N/A | 27.4% | 49.6% | 496 | 66.9 | 59.7% | 55.5% |  | 20.6% | 56.9% | 21.0% | 1.6% | 61.1 | 57.5% |  | 30.2% | 49.4% | 37 months | LVEF>45% |
| CAMEO-DAPA | Dapagliflozin | SGLT2i | Placebo | Placebo | 21 | 67 | 33.3% | 61.0% | 35.0 | 0.0% | 33.3% | 66.7% | 0.0% | 71 | 66.7% | 38.1% | 28.6% | N/A | 17 | 67 | 35.3% | 63.0% | 34.5 | 0.0% | 29.4% | 70.6% | 0.0% | 73.0 | 58.8% | 35.3% | 5.9% | N/A | 24 weeks | LVEF≥50% |
| CHARM-Preserved | Candesartan | ARB | Placebo | Placebo | 1514 | 67.2 | 60.8% | 54.0% | 29.3 | 0 | 61.5% | 36.7% | 1.8% |  | 65.0% | 29.0% | 28.7% | 45.0% | 1509 | 67.1 | 59.0% | 54.1% | 29.0 | 0.0% | 60.0% | 38.7% | 1.3% |  | 63.6% | 29.3% | 28.0% | 43.7% | 36.6 months | LVEF >40% |
| DELIVER | Dapagliflozin | SGLT2i | Placebo | Placebo | 3131 | 71.8 | 56.4% | 54.0% | 29.8 | 0 | 73.90% | 25.80% | 0.30% | 61 | 88.00% | 56.1% | 44.7% | N/A | 3132 | 71.5 | 55.8% | 54.3% | 29.9 | 0 | 76.60% | 23.10% | 0.30% | 61.0 | 89.3% | 57.3% | 44.9% | N/A | 2.3 years | LVEF>40% |
| DETERMINE-Preserved | Dapagliflozin | SGLT2i | Placebo | Placebo | 253 | 73 | 64.0% | 50.0% | 29.0 | 0 | 81% | 19% | 0 | 57 | N/A | 54.2% | 43.1% | N/A | 251 | 73 | 62.9% | 53.0% | 28.0 | 0 | 86.40% | 13.60% | 0 | 58.0 | N/A | 49.8% | 44.2% | N/A | 16 weeks | LVEF>40% |
| ELANDD | Nebivolol | Beta Blocker | Placebo | Placebo | 57 | 66.5 | 35.1% | 61.9% | 30.3 | 0 | 77.20% | 22.80% | 0 | N/A | 86.00% | N/A | N/A | N/A | 59 | 65.3 | 35.6% | 63.2% | 30.2 | 0 | 78.00% | 22.00% | 0 | N/A | 86.4% | N/A | N/A | N/A | 6 months | LVEF>45% |
| EMPA-VISION | Empagliflozin | SGLT2i | Placebo | Placebo | 18 | 69.1 | 55.6% | 59.1% | 30.6 | 0 | 83.30% | 16.70% | 0 | 72 | 38.90% | N/A | 11.1% | N/A | 18 | 72.1 | 50.0% | 52.6% | 30.8 | 0 | 77.80% | 16.70% | 5.60% | 64.1 | 27.8% | N/A | 11.1% | N/A | 12 weeks | LVEF>50% |
| EMPERIAL-Preserved | Empagliflozin | SGLT2i | Placebo | Placebo | 157 | 74 | 55.4% | 53.0% | 30.1 | 0 | 74.50% | 24.80% | 0 | 54.5 | N/A | 31.8% | 54.8% | N/A | 158 | 75 | 58.2% | 53.0% | 28.8 | 0 | 79.70% | 20.30% | 0 | 58.5 | N/A | 28.5% | 47.5% | N/A | 12 weeks | LVEF>40% |
| EMPEROR-Preserved | Empagliflozin | SGLT2i | Placebo | Placebo | 2997 | 71.8 | 55.4% | 54.3% | 29.8 | 0.10% | 81.10% | 18.40% | 0.30% | 60.6 | 90.80% | 51.5% | 48.9% | N/A | 2991 | 71.9 | 55.3% | 54.3% | 29.9 | <0.1% | 81.90% | 17.80% | 0.30% | 60.6 | 90.4% | 50.6% | 49.2% | N/A | 26.2 months | LVEF>40% |
| EMPULSE | Empagliflozin | SGLT2i | Placebo | Placebo | 115 | Not available | |  |  |  |  |  |  |  |  |  |  |  |  |  |  |  |  |  |  |  |  |  |  |  |  |  | 90 days | LVEF≥50% |
| EXCEED | ipragliflozin | SGLT2i | Placebo | Placebo | 36 | 71.9 | 61.1% | 60.9% | N/A | 83.30% | 16.70% | 0 | 0 | N/A | N/A | N/A | 100.0% | N/A | 32 | 70.3 | 59.4% | 60.4% | N/A | 87.50% | 12.50% | 0 | 0 | N/A | N/A | N/A | 100.0% | N/A | 24 weeks | LVEF≥50% |
| EXSCEL | Exenatide | GLP-1 RA | Placebo | Placebo | 499 |  |  |  |  |  |  |  |  |  |  |  |  |  | 561 |  |  |  |  |  |  |  |  |  |  |  |  |  | 3.2 years | LVEF≥40% |
| FINEARTS-HF | Finerenone | MRA | Placebo | Placebo | 3003 | 71.9 | 54.9% | 52.6% | 29.9 | 0 | 69.30% | 30.10% | 0.60% | 61.9 | 87.90% | 38.8% | 40.5% | 26.1% | 2998 | 72 | 54.1% | 52.5% | 30.0 | 0 | 68.90% | 30.40% | 0.80% | 62.3 | 89.6% | 37.6% | 40.8% | 25.3% | 32 months | LVEF ≥40% |
| FLOW | Semaglutide | GLP-1 RA | Placebo | Placebo | 167 |  |  |  |  |  |  |  |  |  |  |  |  |  | 158 |  |  |  |  |  |  |  |  |  |  |  |  |  | 3.4 years | LVEF≥50% |
| IMPRESS-AF | Spironolactone | MRA | Placebo | Placebo | 125 | 73 | 78.0% | 58.0% | 29.0 | N/A | N/A | N/A | N/A | N/A | N/A | 100.0% | 19.0% | N/A | 125 | 72 | 75.0% | 5800.0% | 30.0 | N/A | N/A | N/A | N/A | N/A | N/A | 100.0% | 17.0% | N/A | 2 years | LVEF ≥55% |
| INDIE-HFpEF | Sodium nitrite | Nitrite | Placebo | Placebo | Cross-over RCT | |  |  |  |  |  |  |  |  |  |  |  |  |  |  |  |  |  |  |  |  |  |  |  |  |  |  | 4 weeks | LVEF≥50% |
| I-PRESERVE | Irbesartan | ARB | Placebo | Placebo | 2067 | 72 | 41.0% | 59.0% | 29.7 | 0 | 21% | 77% | 3% | N/A | 89% | 29.0% | 28.0% | 24.0% | 2061 | 72 | 39.0% | 60.0% | 29.6 | 0 | 22% | 76% | 3% | N/A | 88.0% | 29.0% | 27.0% | 23.0% | 49.5 months | LVEF≥45% |
| J-DHF | Carvedilol | Beta Blocker | Placebo | Placebo | 120 | 73 | 57.5% | 62.0% | 24.2 | 18.33% | 69.17% | 10.83% | 1.67% | 58 | 80% | 50.8% | 27.5% | N/A | 125 | 71 | 58.4% | 63.0% | 24.1 | 18.40% | 75.20% | 4.80% | 1.60% | 58.3 | 80.8% | 45.6% | 33.6% | N/A | 3.2 years | LVEF >40% |
| NEAT-HFpEF | Isosorbide Mononitrate | Nitrate | Placebo | Placebo | Cross-over RCT | |  |  |  |  |  |  |  |  |  |  |  |  |  |  |  |  |  |  |  |  |  |  |  |  |  |  | 12 weeks | LVEF≥50% |
| PARAGLIDE-HF | Sacubitril/valsartan | ARNI | Valsartan | ARB | 233 | 71 | 48.1% | 55.2% | 33.3 | 3.40% | 43.80% | 50.20% | 1.70% | 47.4 | 97.90% | 60.1% | 45.9% | 5.2% | 233 | 72 | 48.1% | 55.7% | 32.7 | 3.90% | 43.30% | 48.10% | 4.30% | 51.1 | 94.0% | 57.1% | 51.1% | 6.4% | 5.9 month | LVEF >40% |
| PARAGON-HF | Sacubitril/valsartan | ARNI | Valsartan | ARB | 2407 | 72.7 | 48.4% | 57.6% | 30.2 | 3.00% | 77.50% | 19.00% | 0.30% | 63 | 95.70% | 32.2% | 43.5% | 23.3% | 2389 | 72.8 | 48.2% | 57.5% | 30.3 | 2.70% | 77.00% | 19.80% | 0.50% | 62.0 | 95.4% | 32.5% | 42.5% | 21.9% | 35 months | LVEF≥45% |
| PARALLAX | Sacubitril/valsartan | ARNI | Placebo | Placebo | 1281 | 72.9 | 49.8% | 56.7% | 30.6 | 0.10% | 67.00% | 32.50% | 0.40% | 62.5 | 96.90% | 54.6% | 44.2% | 23.0% | 1285 | 72.4 | 48.8% | 56.2% | 30.5 | 0.30% | 68.20% | 31.20% | 0.30% | 62.7 | 97.4% | 53.9% | 45.8% | 23.8% | 24 weeks | LVEF >40% |
| PARAMOUNT | Sacubitril/valsartan | ARNI | Valsartan | ARB | 149 | 70.9 | 43.0% | 58.0% | 30.1 | 1% | 81% | 19% | 0 | 67 | 95% | 40.0% | 41.0% | 21.0% | 152 | 71.2 | 44.0% | 58.0% | 29.8 | 1% | 78% | 21% | 0 | 64.0 | 92.0% | 43.0% | 35.0% | 20.0% | 36 weeks | LVEF≥45% |
| PEP-CHF | Perindopril | ACEi | Placebo | Placebo | 424 | 75 | 46.0% | 65.0% | 27.5 | I/II: 77% |  | III/IV: 23% | | N/A | 79% | 19.0% | 21.0% | 27.0% | 426 | 75 | 43.0% | 64.0% | 27.6 | I/II: 74% |  | III/IV: 26% | | N/A | 79.0% | 22.0% | 20.0% | 26.0% | 2.1 years | LVEF≥40% |
| PRESERVED-HF | Dapagliflozin | SGLT2i | Placebo | Placebo | 162 | 69 | 43.2% | 60.0% | 35.1 | 0 | 59.30% | III/IV: 40.1% | | 56 | N/A | 50.6% | 55.6% | N/A | 162 | 71 | 43.2% | 60.0% | 34.6 | 0 | 55.60% | III/IV: 44.4% | | 54.0 | N/A | 54.9% | 56.2% | N/A | 12 weeks | LVEF≥45% |
| RAAM-PEF | Eplerenone | MRA | Placebo | Placebo | 21 | 72.2 | 95.2% | 62.1% | 30.1 | 0 | 66.70% | 33.30% | 0 | N/A | 100% | 14.3% | 61.9% | N/A | 23 | 68.7 | 91.3% | 62.5% | 34.6 | 0 | 52.20% | 47.80% | 0 | N/A | 100.0% | 13.0% | 60.9% | N/A | 28 weeks | LVEF≥50% |
| SELECT | Semaglutide | GLP-1 RA | Placebo | Placebo | 1174 | Not available | |  |  |  |  |  |  |  |  |  |  |  | 1099 |  |  |  |  |  |  |  |  |  |  |  |  |  | 39.8 months | LVEF≥50% |
| STEP-HFpEF | Semaglutide | GLP-1 RA | Placebo | Placebo | 263 | 70 | 43.3% | 57.0% | 37.2 | 0 | 69.60% | III/IV: 30.4% | | N/A | 82.10% | 51.3% | N/A | N/A | 266 | 69 | 44.4% | 57.0% | 36.9 | 0 | 62.8% | III/IV: 37.2% | | N/A | 81.6% | 52.6% | N/A | N/A | 52 weeks | LVEF≥45% |
| STEP-HFpEF DM | Semaglutide | GLP-1 RA | Placebo | Placebo | 310 | 69 | 58.7% | 57.0% | 36.9 | 0 | 71.90% | III/IV: 28.1% | | 70 | 82.30% | 37.7% | 100.0% | N/A | 306 | 70 | 52.6% | 55.0% | 36.9 | 0 | 69.3% | III/IV: 30.7% | | 68.3 | 88.6% | 41.2% | 100.0% | N/A | 57 weeks | LVEF≥45% |
| SUMMIT | Tirzepatide | GLP-1 RA | Placebo | Placebo | 364 | 65.5 | 45.1% | 61.0% | 38.3 | 0 | 72.00% | III/IV: 28.0% | | 64.5 | N/A | 26.1% | 47.8% | N/A | 367 | 65 | 47.3% | 60.6% | 38.2 | 0 | 73.0% | III/IV: 27.0% | | 64.3 | N/A | 24.8% | 48.5% | N/A | 104 weeks | LVEF≥50% |
| SUPPORT | Olmesartan | ARB | Placebo | Placebo | 363 | 66.5 | 70.2% | 63.8% | 24.4 | 0 | 94.20% | 5.50% | 0 | 65.3 | 100% | N/A | 46.6% | N/A | 346 | 65.9 | 71.1% | 63.1% | 24.8 | 0 | 93.40% | 6.40% | 0 | 66.0 | 100.0% | N/A | 53.9% | N/A | 4.4 years | LVEF≥50% |
| TOPCAT | Spironolactone | MRA | Placebo | Placebo | 1722 | 68.7 | 48.4% | 56.0% | 31.0 | 3.30% | 63.30% | 33.00% | 0.40% | 65.3 | N/A | N/A | N/A | N/A | 1723 | 68.7 | 48.5% | 56.0% | 31.0 | 3.10% | 64.10% | 32.10% | 0.50% | 65.5 | N/A | N/A | N/A | N/A | 3.3 years | LVEF≥45% |
| Kasama 2005 | Candesartan | ARB | Placebo | Placebo | 25 | 66 | 68.0% | 54.0% | N/A | 0 | 64% | 36% | 0 | N/A | N/A | N/A | N/A | N/A | 25 | 67 | 64.0% | 55.0% | N/A | 0 | 68% | 32% | 0 | N/A | N/A | N/A | N/A | N/A | not given | LVEF >40% |
| Kitzman 2010 | Enalapril | ACEi | Placebo | Placebo | 35 | 69 | 20.0% | 65.0% | 30.0 | 0 | 83% | 17% | 0 | N/A | 71% | N/A | 9.0% | N/A | 36 | 70 | 11.0% | 65.0% | 30.0 | 0 | 75% | 25% | 0 | N/A | 75.0% | N/A | 17.0% | N/A | 12 months | LVEF ≥50% |
| Kurrelmeyer 2014 | Spironolactone | MRA | Placebo | Placebo | 24 | 66.3 | 0.0% | 62.5% | 29.4 | 0 | 33% | 67% | 0 | N/A | 87.50% | 25.0% | 50.0% | N/A | 24 | 76.4 | 0.0% | 62.9% | 26.3 | 0 | 42% | 58% | 0 | N/A | 79.2% | 25.0% | 25.0% | N/A | 6 months | LVEF ≥50% |
| Mittal 2017 | Metoprolol | Beta Blocker | Placebo | Placebo | 20 | 55.2 | 45.0% | 62.9% | N/A | 0 | 55% | 45% | 0 | N/A | N/A | N/A | N/A | N/A | 20 | 57.2 | 50.0% | 62.1% | N/A | 0 | 65% | 35% | 0 | N/A | N/A | N/A | N/A | N/A | 12 weeks | LVEF ≥50% |
| Sheng 2023 | Sacubitril/valsartan | ARNI | Placebo | Placebo | 80 | 55.8 | 41.3% | N/A | 24.2 | 0 | 58.75% | 26.25% | 15% | N/A | 47.50% | N/A | 11.3% | N/A | 80 | 55.85 | 38.8% | N/A | 23.5 | 0 | 56.25% | 30.00% | 13.75% | N/A | 40.0% | N/A | 18.8% | N/A | 6 months | LVEF ≥50% |
| Upadhya 2017 | Spironolactone | MRA | Placebo | Placebo | 42 | 70 | 19.0% | 62.6% | 31.5 | 0 | 29% | 64% | 0 | N/A | 83% | N/A | 17.0% | N/A | 38 | 72 | 21.0% | 62.0% | 32.4 | 0 | 26% | 63% | 0 | N/A | 92.0% | N/A | 29.0% | N/A | 9 months | LVEF ≥50% |
| Zi 2003 | Quinapril | ACEi | Placebo | Placebo | 36 | 77 | 38.9% | N/A | N/A | 5.50% | 77.80% | 16.70% | 0 | N/A | 27.80% | 38.9% | 11.1% | N/A | 38 | 78 | 31.6% | N/A | N/A | 0 | 73.70% | 26.30% | 0 | N/A | 31.6% | 31.6% | 18.4% | N/A | 6 months | LVEF ≥40% |

Supplementary Table S2. Assessment of Homogeneity and Consistency Assumptions

| Primary Outcome | Homogeneity | I^2=0%, τ^2<0.0001 |
| --- | --- | --- |
|  | Inconsistency | p=0.8495 |
|  |  |  |
| CV death | Homogeneity | I^2=0%, τ^2<0.0001 |
|  | Inconsistency | p=0.9523 |
|  |  |  |
| All-cause death | Homogeneity | I^2=0%, τ^2<0.0001 |
|  | Inconsistency | p=0.8761 |
|  |  |  |
| Worsening HF | Homogeneity | I^2=0%, τ^2<0.0001 |
|  | Inconsistency | p=0.6266 |
|  |  |  |
| KCCQ-CSS | Homogeneity | I^2=0%, τ^2<0.0001 |
|  | Inconsistency | p=0.5144 |
|  |  |  |
| 6MWT | Homogeneity | I^2=28%, τ^2<16.1252 |
|  | Inconsistency | p=0.1782 |
|  |  |  |
| NT-proBNP | Homogeneity | I^2=0%, τ^2<0.0001 |
|  | Inconsistency | p=0.8763 |

Supplementary Table S3. Ranking Probabilities among All Explored Outcomes

| Primary Outcome | P-score |  | Worsening HF | P-score |  | 6MWT | P-score |  | NT-proBNP | P-score |
| --- | --- | --- | --- | --- | --- | --- | --- | --- | --- | --- |
| GLP-1 RA | 0.8705 |  | GLP-1 RA | 0.9662 |  | ARNI | 0.8920 |  | ARNI | 0.8920 |
| SGLT2i | 0.7084 |  | SGLT2i | 0.7252 |  | MRA | 0.7737 |  | MRA | 0.7737 |
| MRA | 0.4749 |  | Digoxin | 0.6054 |  | GLP-1 RA | 0.6117 |  | GLP-1 RA | 0.6117 |
| Digoxin | 0.4320 |  | MRA | 0.5220 |  | SGLT2i | 0.3598 |  | SGLT2i | 0.3598 |
| Beta Blocker | 0.4173 |  | ACEi | 0.3732 |  | ARB | 0.2356 |  | ARB | 0.2356 |
| Placebo | 0.0967 |  | ARB | 0.2283 |  | Placebo | 0.1272 |  | Placebo | 0.1272 |
|  |  |  | Placebo | 0.0796 |  |  |  |  |  |  |
|  |  |  |  |  |  |  |  |  |  |  |
|  |  |  |  |  |  |  |  |  |  |  |
| CV Death | P-score |  | All-Cause Mortality | P-score |  | KCCQ-CSS | P-score |  |  |  |
| SGLT2i | 0.7063 |  | MRA | 0.7238 |  | GLP-1 RA | 0.9897 |  |  |  |
| GLP-1 RA | 0.6650 |  | GLP-1 RA | 0.7116 |  | MRA | 0.8430 |  |  |  |
| MRA | 0.6212 |  | ARNI | 0.5471 |  | SGLT2i | 0.5565 |  |  |  |
| ARNI | 0.5362 |  | SGLT2i | 0.5297 |  | Nitrite | 0.4550 |  |  |  |
| ACEi | 0.4440 |  | Digoxin | 0.4530 |  | ARNI | 0.3712 |  |  |  |
| Digoxin | 0.3822 |  | ARB | 0.4136 |  | Placebo | 0.1886 |  |  |  |
| ARB | 0.3530 |  | Placebo | 0.3455 |  | ARB | 0.0860 |  |  |  |
| Placebo | 0.2922 |  | ACEi | 0.2756 |  |  |  |  |  |  |

Supplementary S4. GRADE Evidence Profile for Primary Outcome for Each Network Treatment Comparison

| Comparison | HR | Certainty | Reasons for downgrading |
| --- | --- | --- | --- |
| SGLT2i vs Placebo | 0.79 [0.70–0.91] | High |  |
| GLP-1 RA vs Placebo | 0.73 [0.61–0.88] | Moderate | Indirectness |
| MRA vs Placebo | 0.86 [0.78–0.95] | High |  |
| Beta Blocker vs Placebo | 0.90 [0.55–1.49] | Low | Risk of bias; Imprecision |
| Digoxin vs Placebo | 0.88 [0.70–1.11] | Low | Risk of bias; Imprecision |
| SGLT2i vs GLP-1 RA | 1.08 [0.86–1.36] | Low | Indirectness; Imprecision |
| SGLT2i vs MRA | 0.92 [0.78–1.09] | Moderate | Imprecision |

Supplementary Table S5. Comparison of Existing Systematic Reviews and the Present Network Meta-analysis

| Study | Population | Treatments | Design | Primary outcomes | Key findings | Difference vs our study |
| --- | --- | --- | --- | --- | --- | --- |
| Lin 2021 (Frontiers in Pharmacology) | LVEF ≥40% | ACEI, ARB, ARNI, β-blocker, MRA, digoxin, sildenafil, vericiguat | Systematic review + frequentist NMA (14 RCTs) | All-cause mortality; CV mortality; HF hospitalization | No therapy reduced mortality; ARNI/ACEI reduced HF hospitalization vs placebo. | Did not include SGLT2i and GLP-1 RA; Did not evaluate patient-centered and functional outcomes |
| Lin 2022 (Cardiovascular Diabetology) | LVEF ≥40% | ACEI, ARB, ARNI, β-blocker, MRA, digoxin, vericiguat, SGLT2i | Updated systematic review + NMA (15 RCTs) | All-cause mortality; cardiac death; HF hospitalization | ACEI/ARNI/SGLT2i reduced HF hospitalization; SGLT2i best for worsening HF. | Did not include GLP-1 RA; Did not evaluate patient-centered and functional outcomes |
| Xiang 2022 (JAMA Network Open) | LVEF ≥40% | ACEI/ARB, ARNI, β-blocker, MRA, SGLT2i | Bayesian NMA of drug classes & combinations (19 RCTs) | First HF hospitalization; all-cause mortality; CV mortality | SGLT2i, ARNI, MRA reduced HF hospitalization; no mortality benefit. | Did not include GLP-1 RA and digoxin; Did not evaluate patient-centered and functional outcomes |
| Zafeiropoulos 2024 (JACC: HF) | LVEF ≥40% | RAS inhibitors, ARNI, β-blocker, MRA, SGLT2i, digoxin, vericiguat | Systematic review + component NMA (13 RCTs) | CV death + first HF hospitalization | ARNI, MRA, SGLT2i reduced composite outcome; combo therapy most effective. | Did not include GLP-1 RA; focuses on combo GDMT; Did not evaluate patient-centered and functional outcomes |

Supplementary Table S6. PRISMA NMA Checklist of Items to Include When Reporting A Systematic Review Involving a Network Meta-analysis

| **Section/Topic** | **Item #** | **Checklist Item** | **Reported on Page #** |
| --- | --- | --- | --- |
| **TITLE** |  |  |  |
| Title | 1 | Identify the report as a systematic review *incorporating a network meta-analysis (or related form of meta-analysis).* | Page 1 |
|  |  |  |  |
| **ABSTRACT** |  |  |  |
| Structured summary | 2 | Provide a structured summary including, as applicable:  **Background:** main objectives  **Methods:** data sources; study eligibility criteria, participants, and interventions; study appraisal; and *synthesis methods, such as network meta-analysis.*  **Results:** number of studies and participants identified; summary estimates with corresponding confidence/credible intervals; *treatment rankings may also be discussed. Authors may choose to summarize pairwise comparisons against a chosen treatment included in their analyses for brevity.*  **Discussion/Conclusions:** limitations; conclusions and implications of findings.  **Other:** primary source of funding; systematic review registration number with registry name. | Page 2 |
|  |  |  |  |
| **INTRODUCTION** |  |  |  |
| Rationale | 3 | Describe the rationale for the review in the context of what is already known*, including mention of why a network meta-analysis has been conducted.* | Page 3 |
| Objectives | 4 | Provide an explicit statement of questions being addressed, with reference to participants, interventions, comparisons, outcomes, and study design (PICOS). | Page 3, 4 |
|  |  |  |  |
| **METHODS** |  |  |  |
| Protocol and registration | 5 | Indicate whether a review protocol exists and if and where it can be accessed (e.g., Web address); and, if available, provide registration information, including registration number. | Page 6 |
| Eligibility criteria | 6 | Specify study characteristics (e.g., PICOS, length of follow-up) and report characteristics (e.g., years considered, language, publication status) used as criteria for eligibility, giving rationale. *Clearly describe eligible treatments included in the treatment network, and note whether any have been clustered or merged into the same node (with justification).* | Page 6 |
| Information sources | 7 | Describe all information sources (e.g., databases with dates of coverage, contact with study authors to identify additional studies) in the search and date last searched. | Page 6 |
| Search | 8 | Present full electronic search strategy for at least one database, including any limits used, such that it could be repeated. | Supplementary Appendix |
| Study selection | 9 | State the process for selecting studies (i.e., screening, eligibility, included in systematic review, and, if applicable, included in the meta-analysis). | Page 6, 7 |
| Data collection process | 10 | Describe method of data extraction from reports (e.g., piloted forms, independently, in duplicate) and any processes for obtaining and confirming data from investigators. | Page 6, 7 |
| Data items | 11 | List and define all variables for which data were sought (e.g., PICOS, funding sources) and any assumptions and simplifications made. | Page 6, 7 |
| **Geometry of the network** | **S1** | Describe methods used to explore the geometry of the treatment network under study and potential biases related to it. This should include how the evidence base has been graphically summarized for presentation, and what characteristics were compiled and used to describe the evidence base to readers. | Page 7 |
| Risk of bias within individual studies | 12 | Describe methods used for assessing risk of bias of individual studies (including specification of whether this was done at the study or outcome level), and how this information is to be used in any data synthesis. | Page 6, 7 |
| Summary measures | 13 | State the principal summary measures (e.g., risk ratio, difference in means). *Also describe the use of additional summary measures assessed, such as treatment rankings and surface under the cumulative ranking curve (SUCRA) values, as well as modified approaches used to present summary findings from meta-analyses.* | Page 7 |
| Planned methods of analysis | 14 | Describe the methods of handling data and combining results of studies for each network meta-analysis. This should include, but not be limited to:   - *Handling of multi-arm trials;* - *Selection of variance structure;* - *Selection of prior distributions in Bayesian analyses; and* - *Assessment of model fit.* | Page 7 |
| **Assessment of Inconsistency** | **S2** | Describe the statistical methods used to evaluate the agreement of direct and indirect evidence in the treatment network(s) studied. Describe efforts taken to address its presence when found. | Page 8 |
| Risk of bias across studies | 15 | Specify any assessment of risk of bias that may affect the cumulative evidence (e.g., publication bias, selective reporting within studies). | Page 8 |
| Additional analyses | 16 | Describe methods of additional analyses if done, indicating which were pre-specified. This may include, but not be limited to, the following:   - Sensitivity or subgroup analyses; - Meta-regression analyses; - *Alternative formulations of the treatment network; and* - *Use of alternative prior distributions for Bayesian analyses (if applicable).* | Page 11 |
|  |  |  |  |
| **RESULTS†** |  |  |  |
| Study selection | 17 | Give numbers of studies screened, assessed for eligibility, and included in the review, with reasons for exclusions at each stage, ideally with a flow diagram. | Page 8, Figure 1 |
| **Presentation of network structure** | **S3** | Provide a network graph of the included studies to enable visualization of the geometry of the treatment network. | Supplementary Figure S2 |
| **Summary of network geometry** | **S4** | Provide a brief overview of characteristics of the treatment network. This may include commentary on the abundance of trials and randomized patients for the different interventions and pairwise comparisons in the network, gaps of evidence in the treatment network, and potential biases reflected by the network structure. | Page 8 |
| Study characteristics | 18 | For each study, present characteristics for which data were extracted (e.g., study size, PICOS, follow-up period) and provide the citations. | Supplementary Table S1 |
| Risk of bias within studies | 19 | Present data on risk of bias of each study and, if available, any outcome level assessment. | Supplementary Figure S3 |
| Results of individual studies | 20 | For all outcomes considered (benefits or harms), present, for each study: 1) simple summary data for each intervention group, and 2) effect estimates and confidence intervals. *Modified approaches may be needed to deal with information from larger networks.* | Supplementary Table S1 |
| Synthesis of results | 21 | Present results of each meta-analysis done, including confidence/credible intervals. *In larger networks, authors may focus on comparisons versus a particular comparator (e.g. placebo or standard care), with full findings presented in an appendix. League tables and forest plots may be considered to summarize pairwise comparisons.* If additional summary measures were explored (such as treatment rankings), these should also be presented. | Page 8-11, Supplementary Table S3 |
| **Exploration for inconsistency** | **S5** | Describe results from investigations of inconsistency. This may include such information as measures of model fit to compare consistency and inconsistency models, *P* values from statistical tests, or summary of inconsistency estimates from different parts of the treatment network. | Page 8 |
| Risk of bias across studies | 22 | Present results of any assessment of risk of bias across studies for the evidence base being studied. | Page 8, Supplementary Figure S3 |
| Results of additional analyses | 23 | Give results of additional analyses, if done (e.g., sensitivity or subgroup analyses, meta-regression analyses*, alternative network geometries studied, alternative choice of prior distributions for Bayesian analyses,* and so forth). | Page 11 |
|  |  |  |  |
| **DISCUSSION** |  |  |  |
| Summary of evidence | 24 | Summarize the main findings, including the strength of evidence for each main outcome; consider their relevance to key groups (e.g., healthcare providers, users, and policy-makers). | Page 12-14 |
| Limitations | 25 | Discuss limitations at study and outcome level (e.g., risk of bias), and at review level (e.g., incomplete retrieval of identified research, reporting bias). *Comment on the validity of the assumptions, such as transitivity and consistency. Comment on any concerns regarding network geometry (e.g., avoidance of certain comparisons).* | Page 15 |
| Conclusions | 26 | Provide a general interpretation of the results in the context of other evidence, and implications for future research. | Page 16 |
|  |  |  |  |
| **FUNDING** |  |  |  |
| Funding | 27 | Describe sources of funding for the systematic review and other support (e.g., supply of data); role of funders for the systematic review. This should also include information regarding whether funding has been received from manufacturers of treatments in the network and/or whether some of the authors are content experts with professional conflicts of interest that could affect use of treatments in the network. | N/A |

Supplementary Table S7. Effects of Pharmacological Therapies on Stroke and Atrial Fibrillation in Patients with HFpEF

| Study name | Intervention | Comparator | Risk Ratio for Stroke | Risk Ratio for Atrial Fibrillation |
| --- | --- | --- | --- | --- |
| Ancillary-DIG | Digoxin | Placebo | 1.13 [0.64–2.01] |  |
| CHARM-Preserved | ARB | Placebo | 0.92 [0.65–1.30] |  |
| DELIVER | SGLT2i | Placebo | 1.10 [0.78–1.56] | 1.21 [0.83–1.78] |
| EMPERIAL-Preserved | SGLT2i | Placebo | 0.00 [-inf–inf] |  |
| EXSCEL | GLP-1 RA | Placebo |  | 1.22 [0.96–1.54] |
| FINEARTS-HF | MRA | Placebo |  | 1.04 [0.76–1.43] |
| I-PRESERVE | ARB | Placebo | 1.17 [0.85–1.60] | 0.89 [0.64–1.22] |
| PARAGLIDE-HF | ARNI | ARB |  | 0.81 [0.34–1.93] |
| PARAGON-HF | ARNI | ARB | 1.10 [0.67–1.81] | 1.06 [0.92–1.21] |
| PARALLAX | ARNI | Placebo |  | 0.77 [0.37–1.57] |
| PEP-CHF | ARNI | Placebo | 0.57 [0.28–1.18] |  |
| PRESERVED-HF | SGLT2i | Placebo | 0.00 [-inf–inf] |  |
| STEP-HFpEF | GLP-1 RA | Placebo |  | 0.40 [0.13–1.27] |
| STEP-HFpEF DM | GLP-1 RA | Placebo |  | 1.17 [0.53–2.56] |
| SUMMIT | GLP-1 RA | Placebo |  | 1.93 [0.98–3.82] |
| TOPCAT | MRA | Placebo | 0.95 [0.67–1.36] |  |

Supplementary Figure S1. Violin Plots


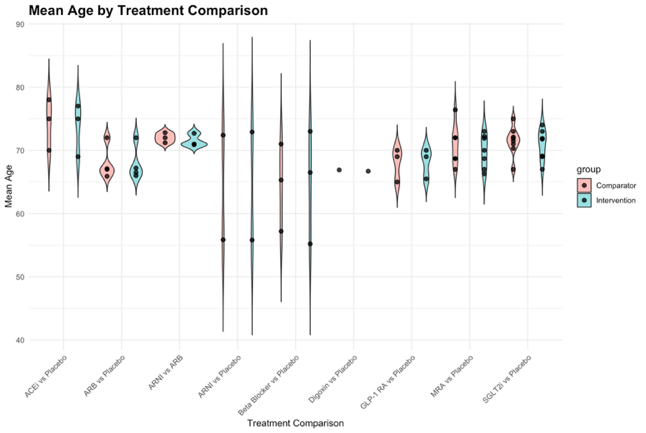

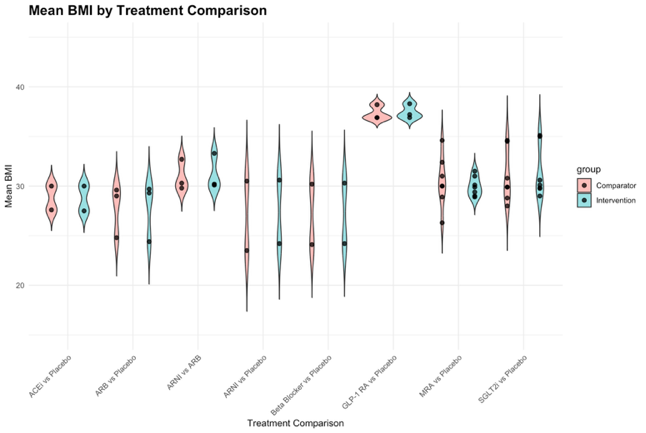

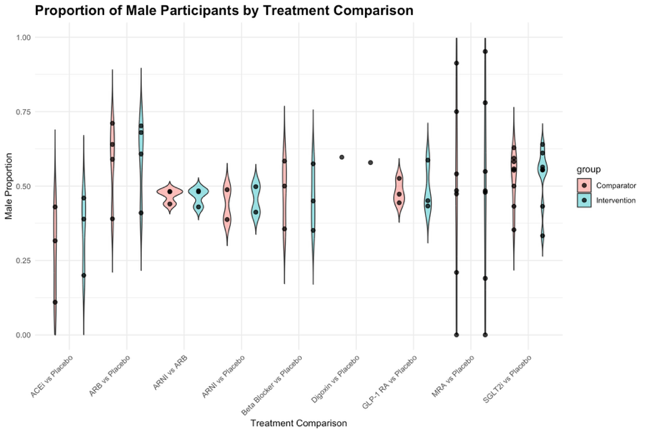

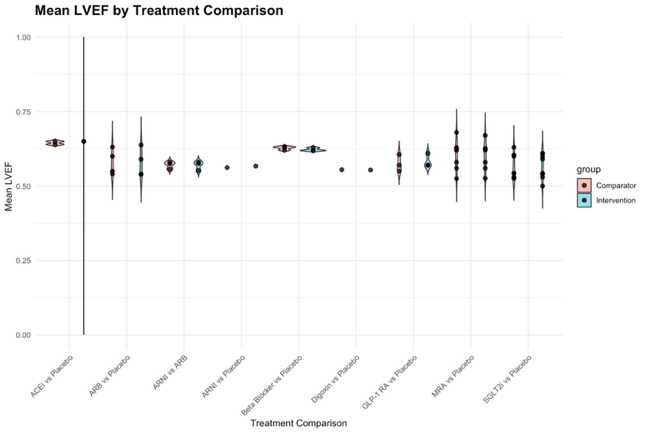


Violin plots showing the distribution for (A) mean age, (B) mean BMI, (C) male%, and (D) mean LVEF acrossed treatment comparisons. ACEi = angiotensin converting enzyme inhibitor; ARB = angiotensin receptor blocker; ARNI = angiotensin receptor-neprilysin inhibitor; BMI = body mass index; CI = confidence interval; HR = hazard ratio; LVEF = left ventricular ejection fraction; MRA = mineralocorticoid receptor antagonist; SGLT2i = sodium-glucose transporter 2 inhibitors; GLP-1 RA = glucagon-like peptide-1 receptor agonist

Supplementary Figure S2. Network Plots

(A)
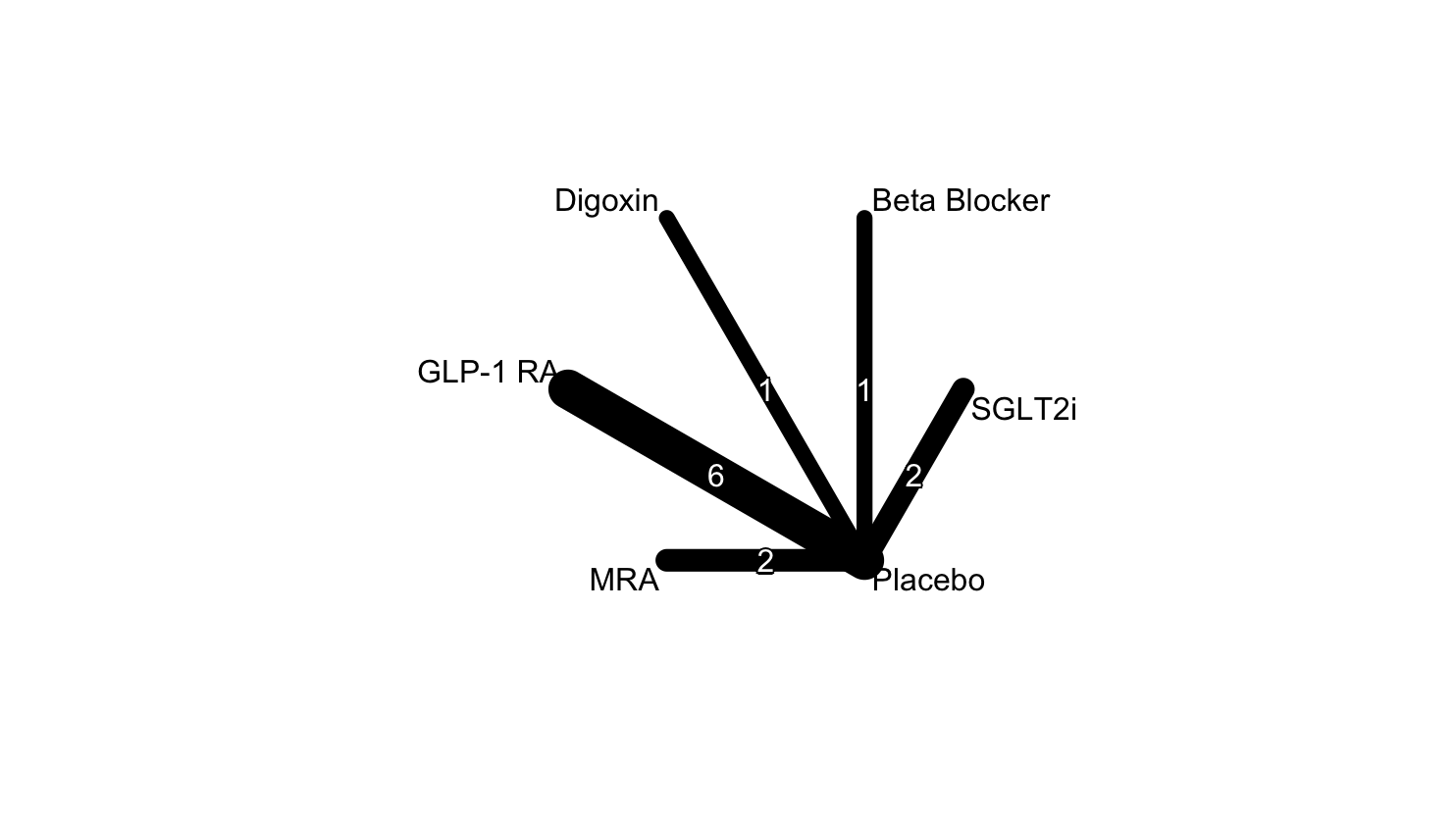
(B)
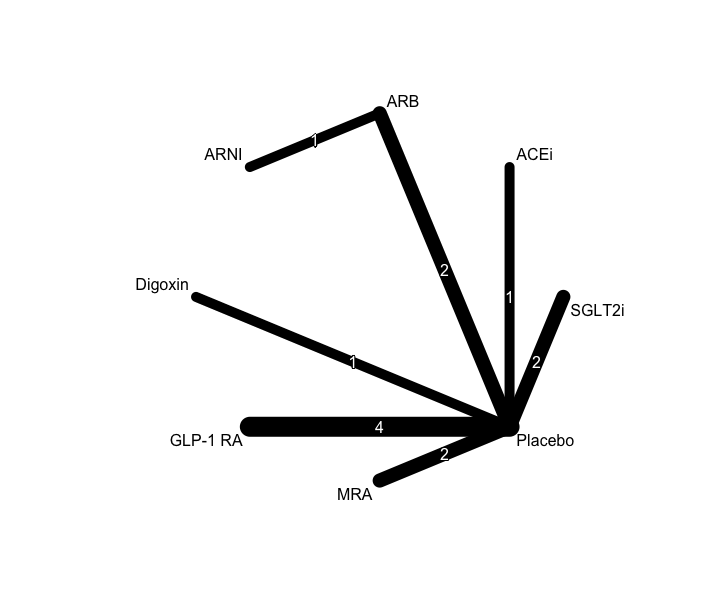
(C)
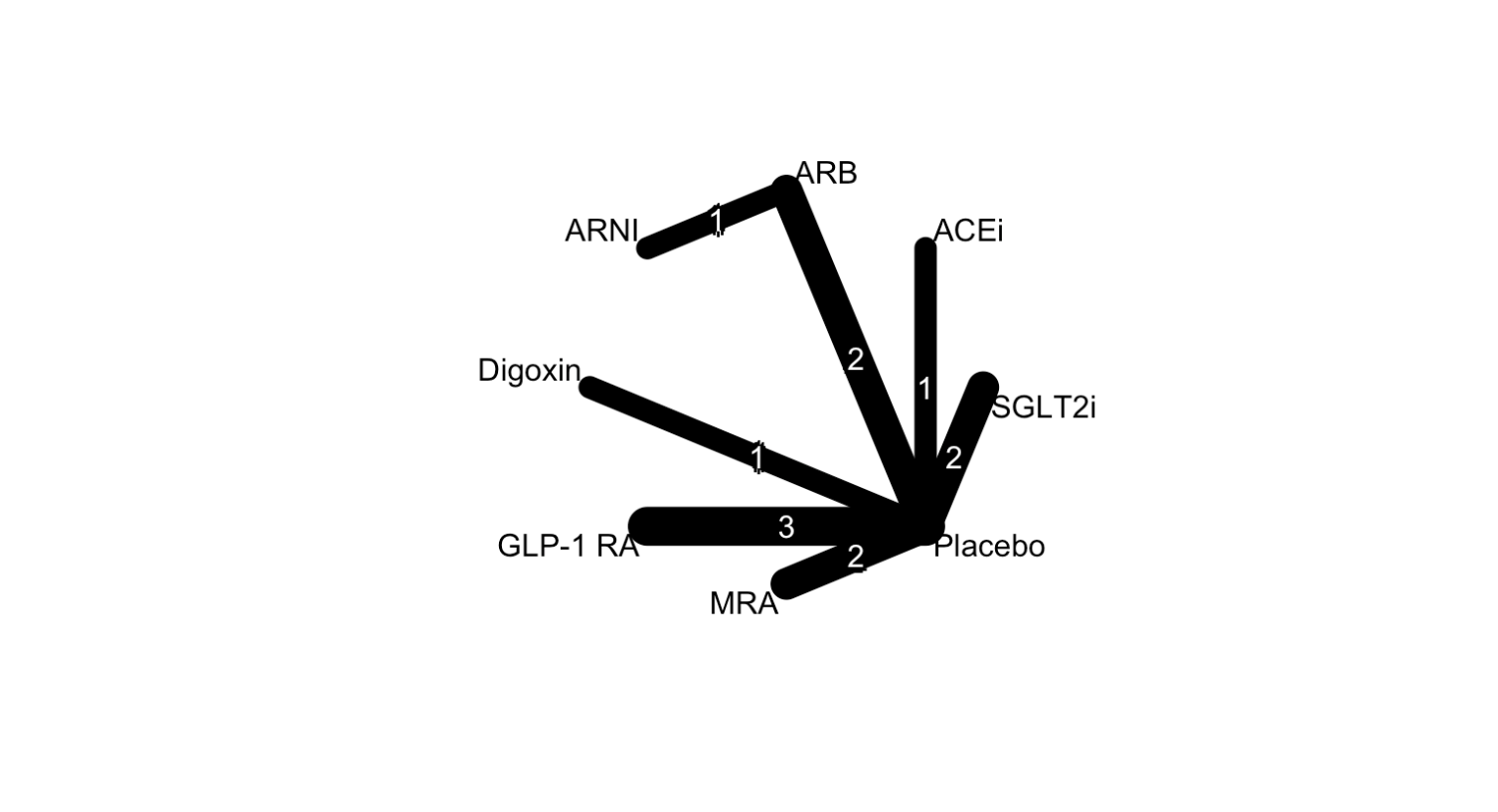
(D)
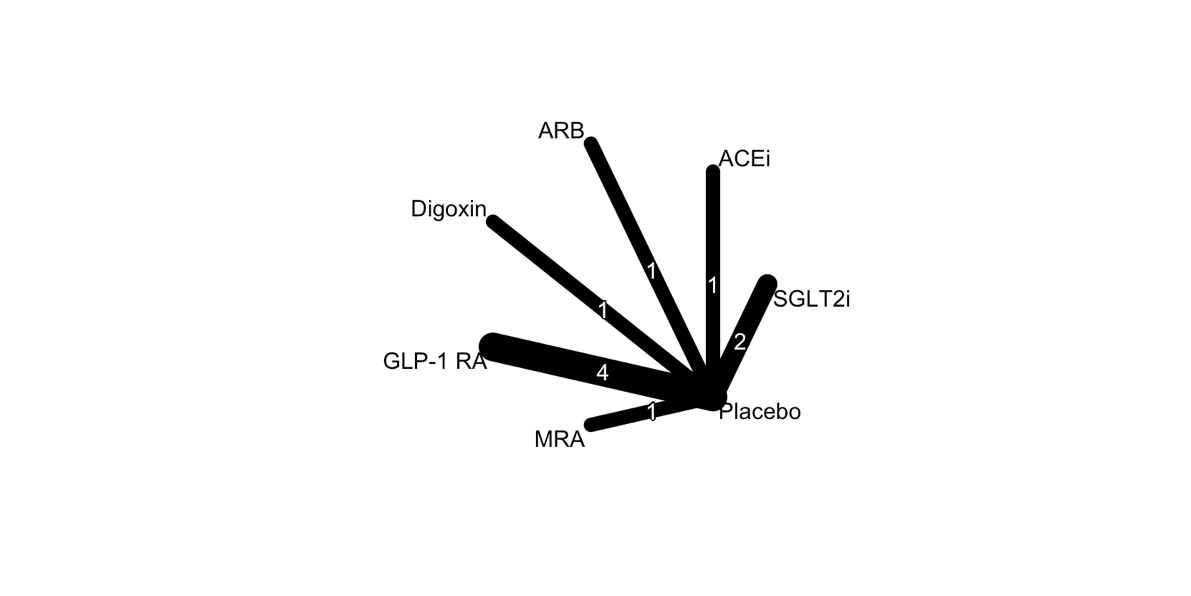
(E)
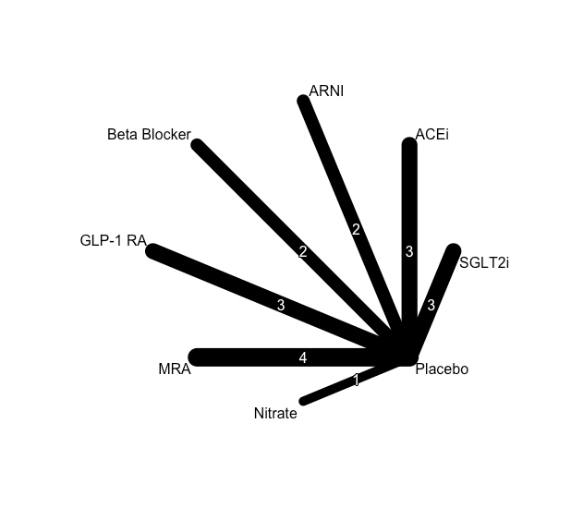
(F)
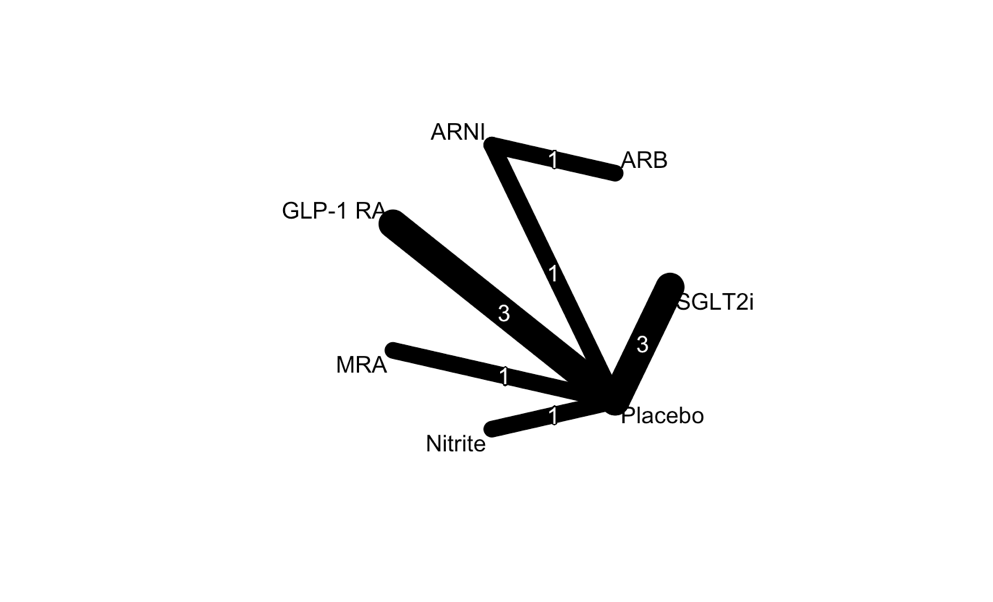
(G)
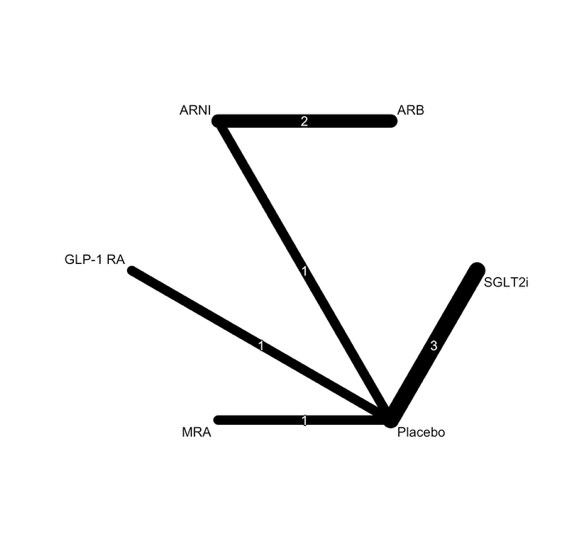
(H)
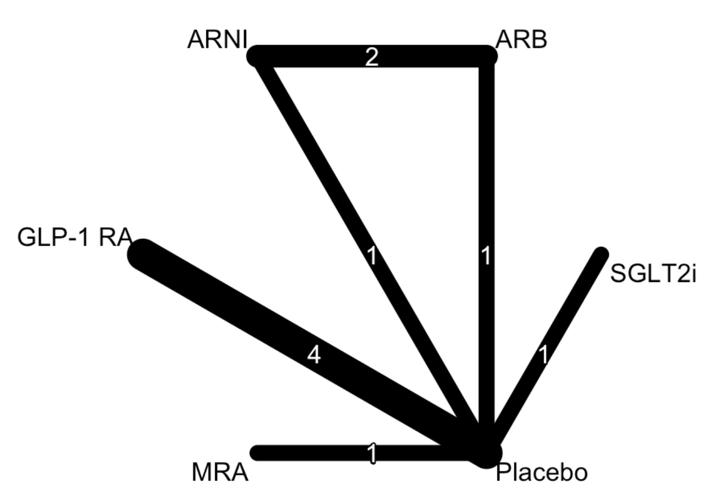
(I)
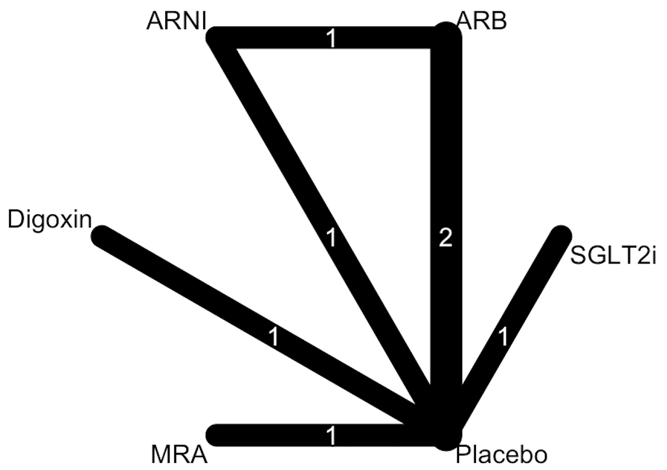


Network plots for (A) primary outcome, (B) CV death, (C) all-cause mortality, (D) worsening HF event, (E) 6MWT, (F) KCCQ-CSS, (G) NTproBNP, (H) atrial fibrillation, and (I) stroke. 6MWT = 6-minute walk test; ACEi = angiotensin converting enzyme inhibitor; ARB = angiotensin receptor blocker; ARNI = angiotensin receptor-neprilysin inhibitor; BMI = body mass index; CI = confidence interval; HF = heart failure; HR = hazard ratio; KCCQ-CSS = Kansas City Cardiomyopathy Questionnaire Clinical Summary Score; MRA = mineralocorticoid receptor antagonist; NTproBNP = N-terminal pro-B-type natriuretic peptide; SGLT2i = sodium-glucose transporter 2 inhibitors; GLP-1 RA = glucagon-like peptide-1 receptor agonist

Supplementary Figure S3. Risk of Bias Table for Non-Crossover RCTs


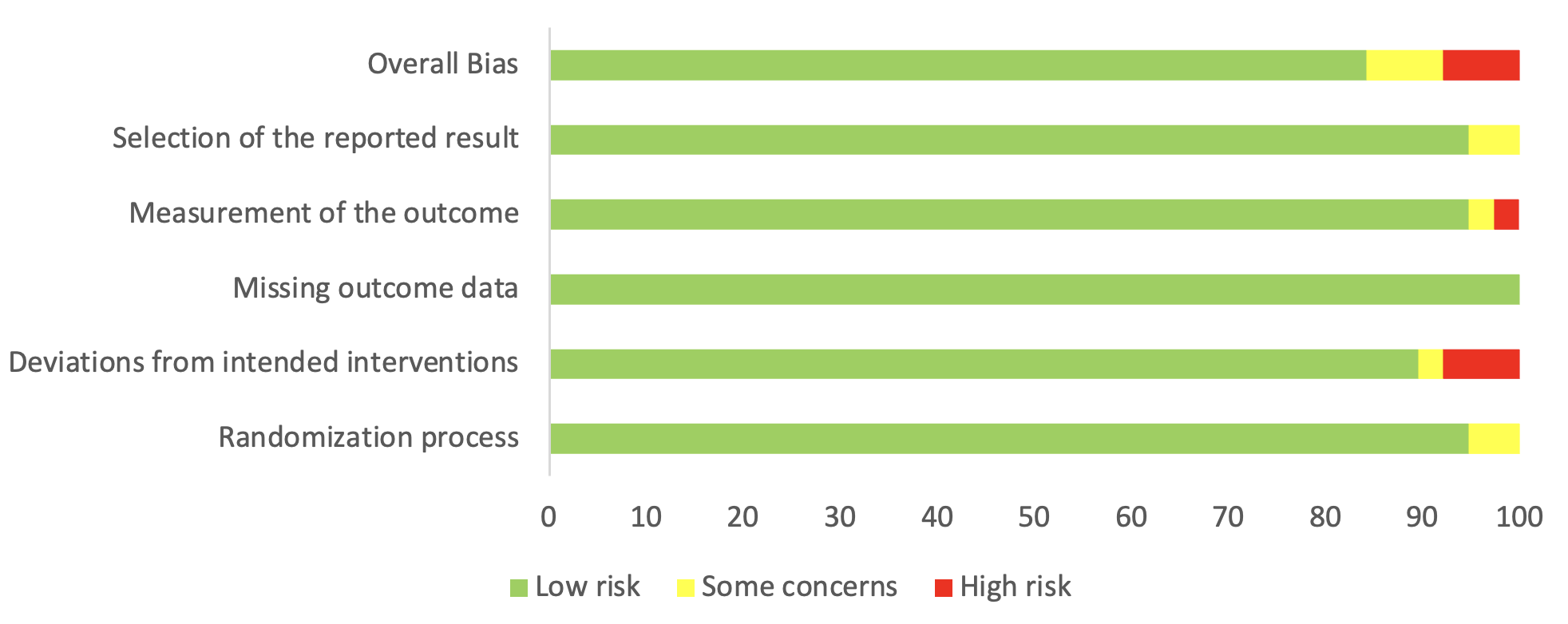


Supplementary Figure S4. Risk of Bias Table for Crossover RCTs


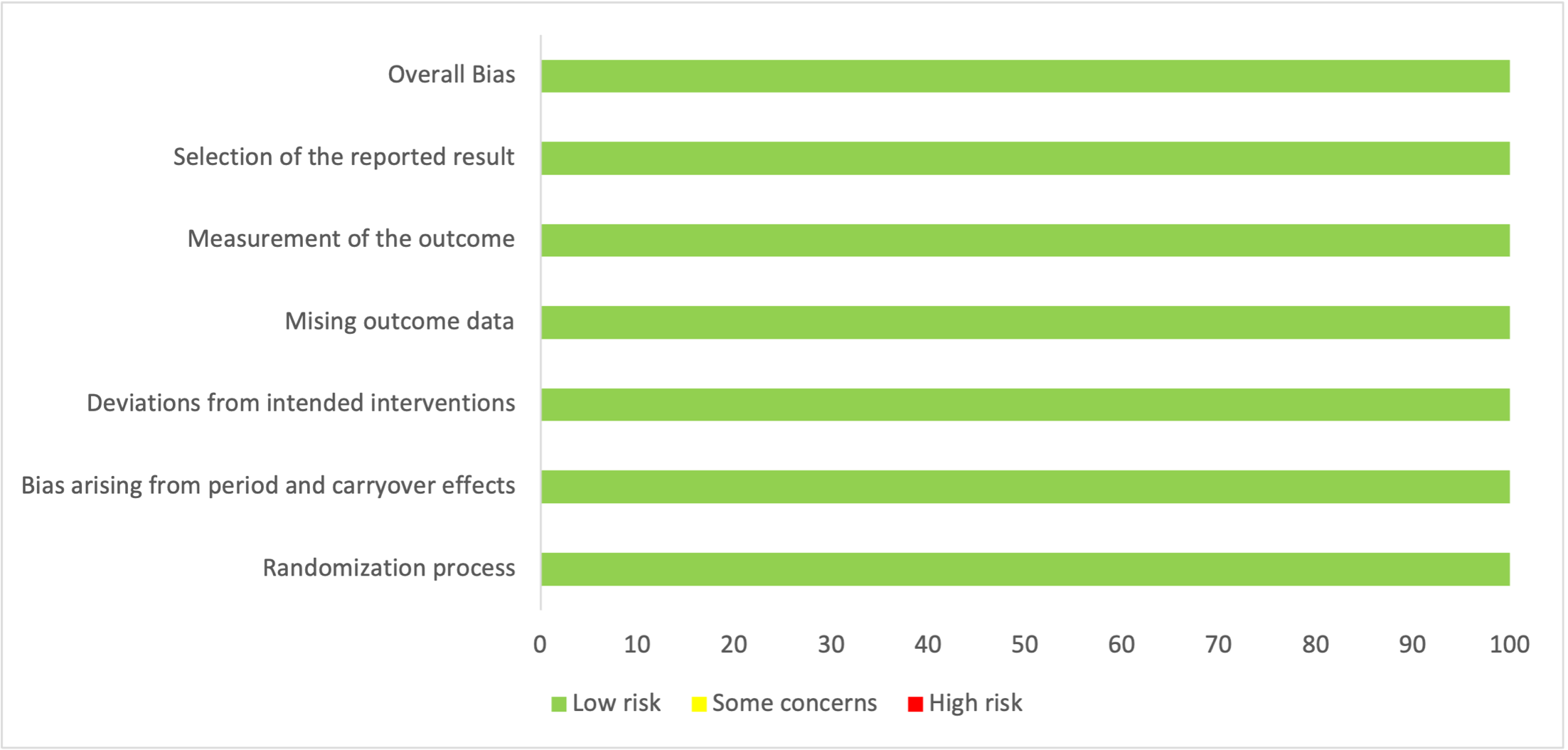


Supplementary Figure S5. Forest Plot of Atrial Fibrillation


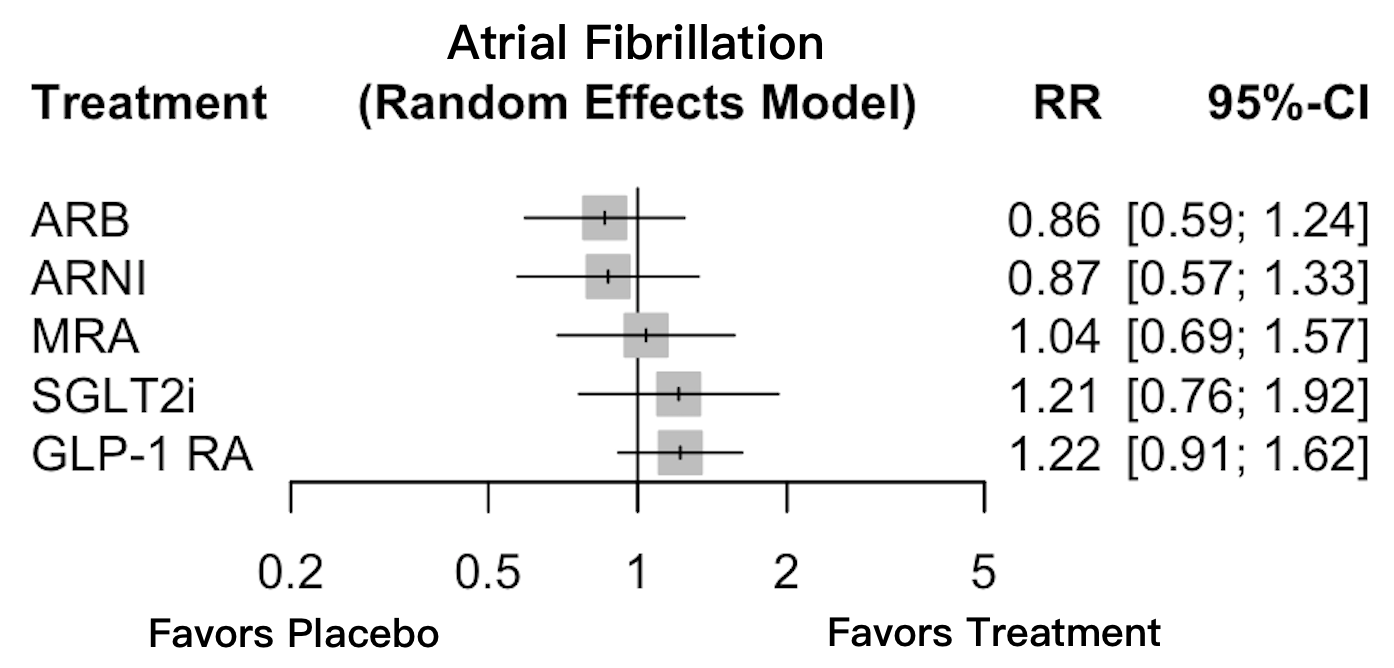


Supplementary Figure S6. Forest Plot of Stroke


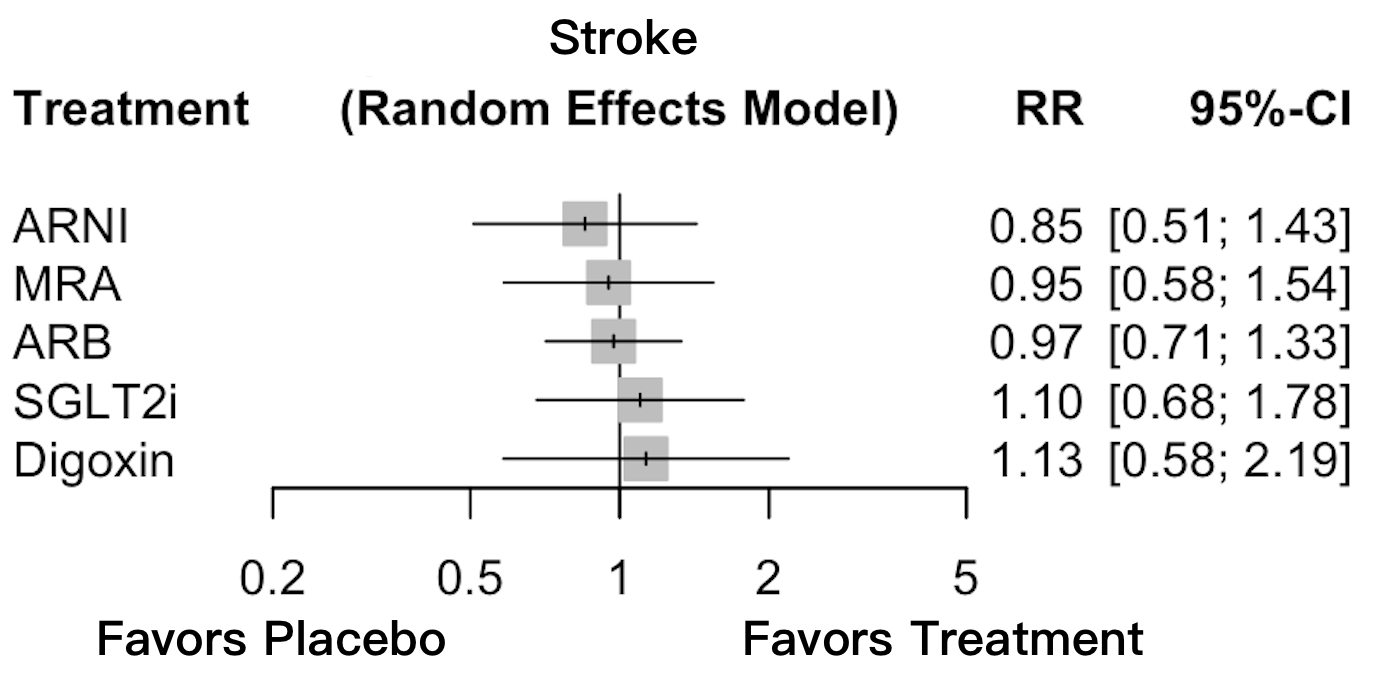

Supplement: Supplementary file 1 — Data S1. Supporting information. [file DOM-28-3137-s001.docx]
